# Core clock gene BMAL1 and RNA-binding protein MEX3A collaboratively regulate Lgr5 expression in intestinal crypt cells

**DOI:** 10.1038/s41598-023-44997-5

**Published:** 2023-10-16

**Authors:** Li-Tzu Cheng, Grace Y. T. Tan, Fang-Pei Chang, Cheng-Kai Wang, Yu-Chi Chou, Pang-Hung Hsu, Wendy W. Hwang-Verslues

**Affiliations:** 1 Genomics Research Center, Academia Sinica, No. 128, Sec. 2, Academia Road, Taipei, 115 Taiwan; 2 Molecular and Cell Biology, Taiwan International Graduate Program, Academia Sinica and Graduate Institute of Life Sciences, National Defense Medical Center, Taipei, Taiwan; 3 Biomedical Translation Research Center, Academia Sinica, Taipei, Taiwan; 4 Department of Bioscience and Biotechnology, National Taiwan Ocean University, Keelung, Taiwan

**Keywords:** Cell biology, Molecular biology

## Abstract

The intestinal epithelium is highly regenerative. Rapidly proliferating LGR5^+^ crypt base columnar (CBC) cells are responsible for epithelial turnover needed to maintain intestinal homeostasis. Upon tissue damage, loss of LGR5^+^ CBCs can be compensated by activation of quiescent +4 intestinal stem cells (ISCs) or early progenitor cells to restore intestinal regeneration. LGR5^+^ CBC self-renewal and ISC conversion to LGR5^+^ cells are regulated by external signals originating from the ISC niche. In contrast, little is known about intrinsic regulatory mechanisms critical for maintenance of LGR5^+^ CBC homeostasis. We found that LGR5 expression in intestinal crypt cells is controlled by the circadian core clock gene BMAL1 and the BMAL1-regulated RNA-binding protein MEX3A. BMAL1 directly activated transcription of *Mex3a*. MEX3A in turn bound to and stabilized *Lgr5* mRNA. *Bmal1* depletion reduced *Mex3a* and *Lgr5* expression and led to increased ferroptosis, which consequently decreased LGR5^+^ CBC numbers and increased the number of crypt cells expressing +4 ISC marker BMI1. Together, these findings reveal a BMAL1-centered intrinsic regulatory pathway that maintains LGR5 expression in the crypt cells and suggest a potential mechanism contributing to ISC homeostasis.

## Introduction

A healthy intestine is essential for digestion, nutrient extraction, absorption and waste removal. The intestinal epithelium is organized into crypt-villus structures. In the crypts, there are two types of intestinal stem cells (ISCs) wedged between Paneth cells which protect and provide growth factors to the ISCs^1^. One type of ISCs are LGR5-expressing (LGR5^+^) crypt base columnar (CBC) stem cells which sit at position +1 to +3 at the crypt base between Paneth cells. The other ISC type are ‘+4’ cells which express BMI1, HOPX and LRIG1^1^. Proliferation of LGR5^+^ CBCs drives the rapid turnover and regeneration of intestinal epithelium to maintain intestinal homeostasis^2,3^. However, fast-proliferating LGR5^+^ CBCs are sensitive to cytotoxic injury resulting from chemotherapy or irradiation (IR). Depletion of LGR5^+^ CBCs by IR or in a toxin receptor-mediated cell knockout mouse model resulted in severe crypt loss, irregular villus structure, and reduced epithelial turnover^4,5^. In contrast to the stress sensitive LGR5^+^ CBCs, +4 cells are more resistant to IR^6^. It has been suggested that these +4 cells can reprogram into LGR5^+^ cells to replenish the crypt base when LGR5^+^ CBCs are depleted due to intestinal injury^7^. In addition, enterocyte progenitors^8^ and occasionally Paneth cells^9^ have also been shown to contribute to replenish the loss of LGR5^+^ CBCs. This robust ISC homeostasis requires interconnected developmental signaling pathways from the ISC niche, including Wnt, Notch, Hedgehog, Bone morphogenetic protein (BMP), Eph-ephrin and EGF^10^, to maintain continuous renewal of the intestinal epithelium.

Several studies have shown that LGR5^+^ CBC self-renewal, fate decisions and ISC conversion are regulated by extrinsic factors^10^. However, recent evidence has indicated that intrinsic signaling factors are also required to maintain the LGR5^+^ CBC pool and ensure adequate regeneration of intestinal epithelium^11^. RNA-binding proteins regulate various aspects of RNA fate and function^12,13^. In the intestine, RNA-binding proteins, including MEX3A, MSI1, HUR and IMP1, facilitate development and repair, as well as maintenance of normal mucosa structures^11,14^. MEX3A expression has been used to identify a subset of slowly dividing LGR5^+^ CBCs^15^. Under chemotherapy-induced intestinal damage, these MEX3A^high^ cells contributed to intestinal epithelium repair and homeostasis^15^. Conversely, MEX3A knockout decreased the number of LGR5^+^ CBCs in the duodenum and delayed organoid formation^11^, indicating that MEX3A is required for survival and stemness functions of LGR5^+^ CBCs. The factors controlling MEX3A expression and the underlying mechanism of MEX3A effect on LGR5^+^ CBCs are unknown.

Despite the increasing recognition that the circadian machinery impacts many physiological functions, it is unclear how the circadian machinery contributes to intestinal homeostasis. A few recent studies have suggested that circadian rhythm affects ISC proliferation and tissue regeneration^16,17^. However, whether the core circadian machinery regulates expression of *Lgr5,* or other ISC genes, is unclear.

Among the core circadian clock genes, BMAL1 is the key transcription factor^18^. BMAL1 forms heterodimers with CLOCK^19^ to transcriptionally regulate genes required for the network of transcription-translation feedback loops (TTFL) that drive circadian rhythms. In the gastrointestinal (GI) tract, it has been shown that BMAL1 deletion inhibited drug export^20^, prevented ghrelin secretion^21^, and promoted glucose uptake^22^. These observations demonstrated an indispensable role of BMAL1 in maintaining GI tract function. BMAL1 has been shown to regulate the timely division of intestinal cells as depletion of *Bmal*1 abolished oscillation of *Wnt3a*, a critical niche component for maintaining LGR5^+^ CBC proliferation, and resulted in disruption of circadian rhythm and cell cycle coupling^17^. In addition, BMAL1 was found to contribute to the inflammation response and cell proliferation during intestinal regeneration^16^. Despite such evidence suggesting that BMAL1 is essential to intestinal cell proliferation and regeneration, the mechanisms by which BMAL1 regulates ISC and intestinal homeostasis remain unclear. In any given mammalian cell or tissue, approximately 5–20% of the transcripts show circadian oscillations^23^, and approximately 35% of the oscillating transcripts are modulated by post-transcriptional control^24^. Several RNA-binding proteins participate in circadian regulation^25–27^. It is possible that the circadian machinery incorporates RNA-binding proteins to mediate post-transcriptional regulation to maintain intestinal homeostasis.

The intestine, particularly the upper small intestine, is the major organ that absorb various chemicals, including nutrients and xenobiotics. Moreover, oncology drugs and radiation therapy for gastrointestinal cancers or para-aortic nodes from gynecological cancers have been reported to cause high toxicity in the small intestine, particularly duodenum^28,29^. Thus, to cope with such frequent stress and toxic insult, the small intestine must rely on tissue regeneration driven by intestinal stem cells to maintain homeostasis and fitness. We found that BMAL1 and MEX3A act together to regulate *Lgr5* expression and may contribute to LGR5^+^ CBC homeostasis in duodenal crypts. In this mechanism, BMAL1 upregulated expression of *Mex3a*, while MEX3A further enhanced LGR5 expression by directly binding and stabilizing *Lgr5* mRNA. *Bmal1* depletion and *Mex3a* downregulation elevated lipid peroxidation level and ferroptosis stress. In mouse models, *Bmal1* knockout in either LGR5^+^ CBCs or in villus and crypt epithelial cells downregulated *Mex3a* and *Lgr5* expression, reduced CBC prevalence and increased the prevalence of BMI1 expressing cells in crypts. These findings suggest a BMAL1-centered intrinsic regulatory pathway contributing to LGR5^+^ CBC homeostasis.

## Results

### BMAL1 maintains LGR5 expression and ISC homeostasis in crypts

**Figure 1:**
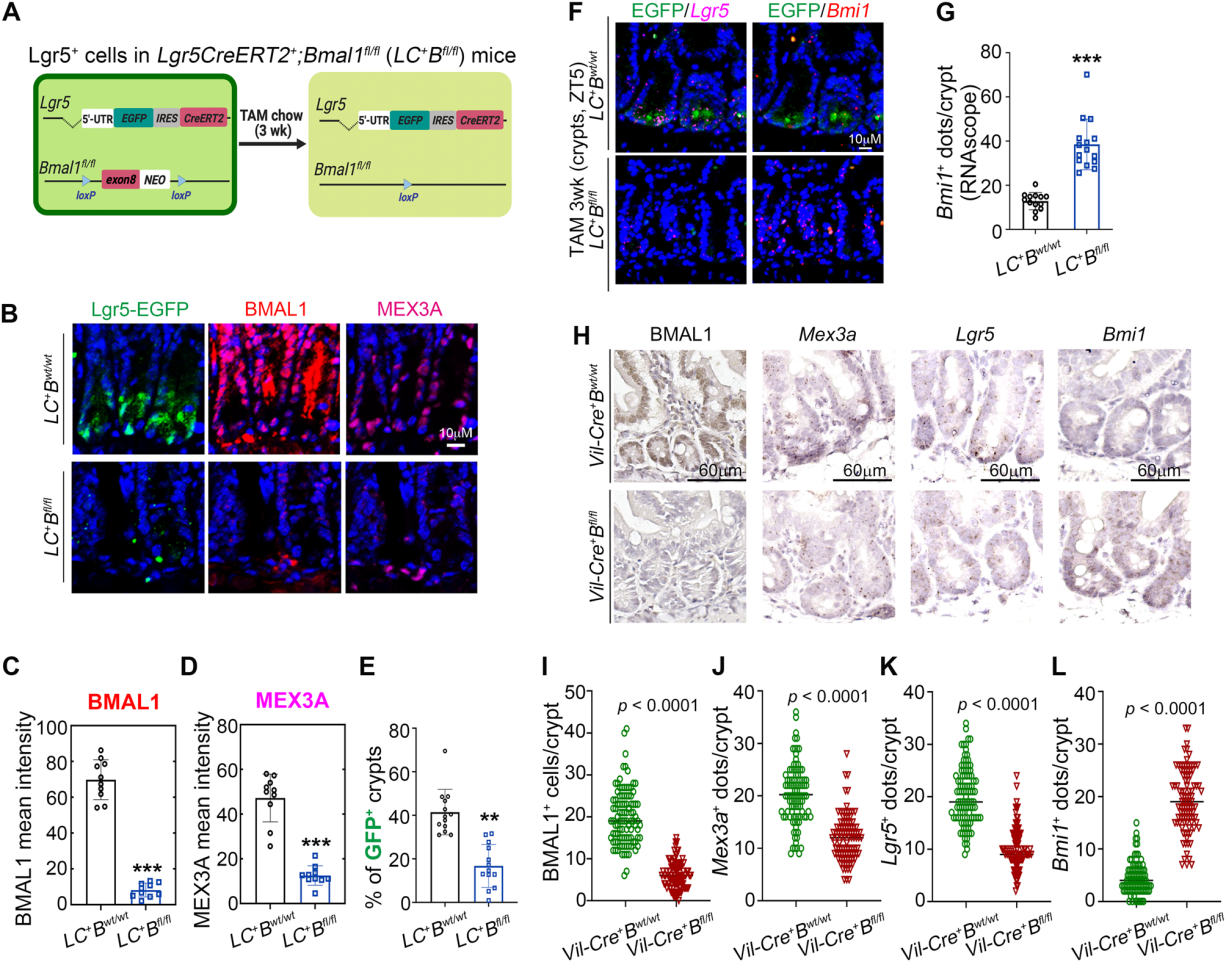
BMAL1 knockout reduces MEX3A expression and decreases LGR5^+^ CBC numbers but increases BMI1^+^ cells in the crypts. (**A**) Diagram of the LGR5^+^ CBC specific BMAL1 knockout mouse model. *Lgr5CreERT2*^+^; *Bmal1*^*wt/wt*^ (*LC*^+^*B*^*wt/wt*^) and *Lgr5CreERT2*^+^; *Bmal1*^*fl/fl*^ (*LC*^+^*B*^*fl/fl*^) male mice were fed with a tamoxifen (TAM) diet for 3 weeks to specifically knockout exon8 of the *Bmal1* gene in LGR5-EGFP^+^ CBCs. (**B**–**E**) Immunofluorescence staining (**B**), quantification of BMAL1 and MEX3A expression in duodenum crypts (**C**, **D**) and prevalence of EGFP^+^ crypts (**E**) from TAM fed male mice at ZT5 (zeitgeber time (ZT), ZT0 corresponds to lights on; ZT12 corresponds to lights off). Scale bar indicates 10 μM. (n = 3 mice, ≥ 50 crypts/n). (**F**) RNAscope analysis of *Lgr5* (pink dots) and *Bmi1* (red dots) mRNA in duodenum crypts of TAM fed *LC*^+^*B*^*wt/wt*^ and *LC*^+^*B*^*fl/fl*^ male mice at ZT5. Scale bar indicates 10 μM (n = 3 mice). (**G**) Quantification of Bmi1 mRNA dots from (**F**). (n = 3 mice, ≥ 50 crypts/n). (**H**–**L**) Immunohistochemistry (IHC) of BMAL1 and RNAscope analysis of *Lgr5*, *Mex3a* and *Bmi1* mRNA in intestinal crypts of *Vil-Cre*^+^*B*^*wt/wt*^ and *Vil-Cre*^+^*B*^*fl/fl*^ male mice at ZT5 (**H**). Scale bar indicates 60 μM. Quantification of BMAL1^+^ cell prevalence (**I**), mRNA expression of *Mex3a* (**J**) and *Lgr5* (**K**), and prevalence of BMI1^+^ cells (**L**) in intestinal crypts of *Vil-Cre*^+^*B*^*wt/wt*^ and *Vil-Cre*^+^*B*^*fl/fl*^ male mice at ZT5. (n = 3 mice, 100 crypts/n). All quantification data are means ± SD, significant differences are based on unpaired T-tests. * *P* < 0.05, ** *P* < 0.01, *** *P* < 0.001, ****P < 0.0001.

To determine the role of BMAL1 in LGR5^+^ CBCs, we established a tamoxifen (TAM) induced-LGR5-expressing cell-specific *Bmal1* knockout mouse model [*Lgr5-Cre*^+^*;Bmal1*^*fl/fl*^ (*LC*^+^*B*^*fl/fl*^)] by crossing *B6.129P2-Lgr5*^*tm1(cre/ERT2)Cle*^*/J* and *B6.129S4(Cg)-Arntl*^*tm1Weit*^*/J* mice (Fig. 1A). In duodenal crypts, *Bmal1* knockout led to a 3.8-fold reduction of MEX3A expression (Fig. 1B–D). Quantification of EGFP^+^ cells at the crypt base found a 2.5-fold decrease of LGR5^+^ CBC-containing crypts in the *LC*^+^*B*^*fl/fl*^ mice compared to the *LC*^+^*B*^*wt/wt*^ control (Fig. 1B and E). It is well established that BMI1^+^  + 4 cells can convert into LGR5-expressing cells upon intestinal damage and LGR5^+^ CBC loss^6,7^. Consistent with this notion, we also found a three-fold increase of *Bmi1* RNA in the crypts of TAM-treated *LC*^+^*B*^*fl/fl*^ mice (Fig. 1F and G).

To confirm that this was not a *LC*^+^*B*^*fl/fl*^ mouse specific phenomenon, we further generated an intestinal villus and crypt epithelial cell-specific *Bmal1* knockout mouse model [*Vil-Cre*^+^*;Bmal1*^*fl/fl*^ (*Vil-Cre*^+^*B*^*fl/fl*^)] by crossing *B6.Cg-Tg(Vil1-cre)1000Gum/J* and *B6.129S4(Cg)-Arntl*^*tm1Weit*^*/J* mice. Consistent with the results from the *LC*^+^*B*^*fl/fl*^ mouse model, *Bmal1* knockout significantly reduced *Mex3a* and *Lgr5* expression (Fig. 1H–K) and increased *Bmi1* expression in intestinal crypts of the *Vil-Cre*^+^*B*^*fl/fl*^ mice (Fig. 1H and L). These observations indicated that, even without external stress or tissue damage, loss of BMAL1 perturbed the homeostasis between LGR5^+^ CBCs and BMI1^+^  +4 cells in the intestine.

**Figure 2:**
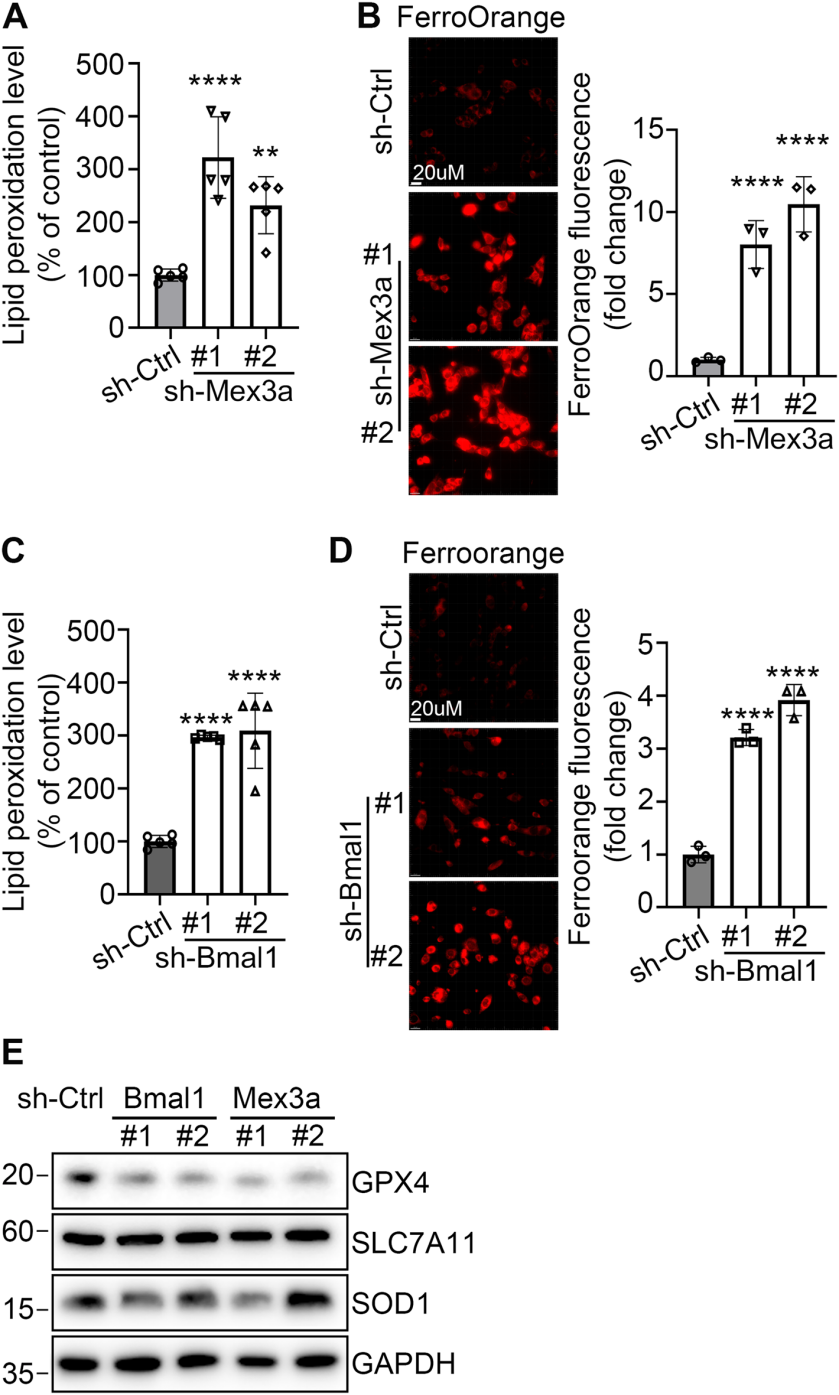
*Mex3a* or *Bmal1* depletion in mIECs elevated lipid peroxidation and ferroptosis stress. (**A**) Lipid peroxidation level in the sh-Ctrl or sh-Mex3a mIEC cells detected by BODIPY-C11 staining. Relative BODIPY-C11 mean fluorescence intensity is presented as percent of control. Three independent experiments were performed and data are means ± SD from one representative experiment (n = 5). Significant differences are based on unpaired T-test (**P < 0.01, ****P < 0.0001). (**B**) Representative images of FerroOrange staining using the sh-Ctrl or sh-Mex3a mIEC cells. Scale bar indicates 20 μm. Relative FerroOrange mean fluorescence intensity is presented as fold change of control. Three independent experiments were performed and data are means ± SD from one representative experiment (n = 3). Significant differences are based on unpaired T-test (****p < 0.0001). (**C**) Lipid peroxidation level in the sh-Ctrl or sh-Bmal1 mIEC cells detected by BODIPY-C11 staining. Data formatting is as described for (**A**). (**D**) Representative images of FerroOrange staining using the sh-Ctrl or sh-Bmal1 mIEC cells. Scale bar indicates 20 μm. Data formatting is as described for (**B**). (**E**) IB of GPX4, SLC7A11 and SOD1 protein expression using sh-Ctrl, sh-Bmal1 or sh-Mex3a knockdown mIEC cells. GAPDH was used as a loading control. Blots shown are from one representative experiment of two replicates. The original blots and additional experiments with similar results are shown in the “Supplementary information” file.

We previously found that human MEX3A inhibited ferroptosis in epithelial ovarian cancer cells^30^. Consistent with those observations, *Mex3a* depletion in an immortalized mouse intestinal epithelial cell line (mIEC) significantly increased lipid peroxidation (Fig. 2A) and intracellular Fe^2+^ levels (Fig. 2B), indicators of ferroptosis. *Bmal1*-downregulated intestinal cells also showed significant increase in both lipid peroxidation (Fig. 2C) and ferroptosis (Fig. 2D), consistent with BMAL1 regulation of *Mex3a* expression. This stress phenotype in *Bmal1* and *Mex3a* downregulated cells was likely due to reduced level of Glutathione peroxidase 4 (GPX4), a phospholipid hydroperoxidase that protects cells against membrane lipid peroxidation^31^ (Fig. 2E). Thus, *Bmal1*-depletion mediated ferroptosis which in turn contributed to the decrease in LGR5^+^ CBCs. Together, these results suggest a critical role of BMAL1 in maintaining the ISC homeostasis.

### BMAL1 and MEX3A co-regulate *Lgr5* expression in intestinal epithelial cells

**Figure 3:**
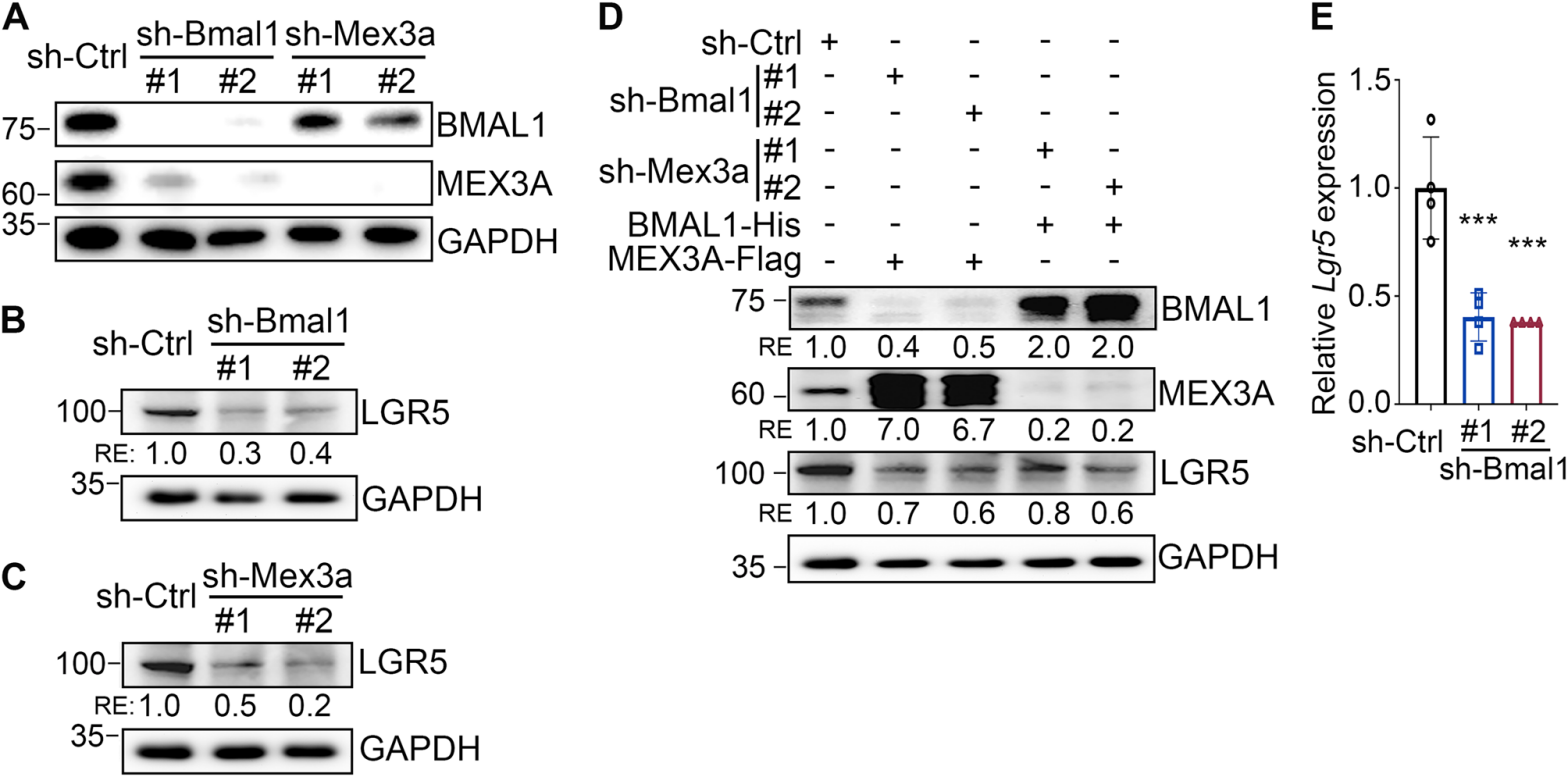
BMAL1 controls LGR5 expression in part by regulating MEX3A. (**A**) Immunoblot of BMAL1, MEX3A and LGR5 in mIEC transduced with sh-Bmal1 or sh-Mex3a lentivirus. GAPDH was used as an internal control. Blots shown are from one representative experiment of three independent experiments. The original blots and additional experiments with similar results are shown in the “Supplementary information” file. (**B**, **C**) Immunoblot of LGR5 in sh-Bmal1 (**B**) or sh-Mex3a (**C**) transduced mIEC. GAPDH was used as an internal control. Blots shown are from one representative experiment of two independent experiments. Numbers below the blot are the quantification of band intensity relative to the sh-Ctrl. The original blots and additional experiments with similar results are shown in the “Supplementary information” file. (**D**) Immunoblot of BMAL1, MEX3A and LGR5 in sh-Bmal1 or sh-Mex3a transduced mIEC cells with MEX3A or BMAL1 overexpression, respectively. GAPDH was used as an internal control. Blots shown are from one representative experiment of two independent experiments. Numbers below each blot are the quantification of band intensity relative to the sh-Ctrl control. The original blots and additional experiments with similar results are shown in the “Supplementary information” file. (**E**) qRT-PCR of *Lgr5* in mIEC cells transduced with sh-Ctrl or sh-Bmal1 lentiviruses. Three independent experiments were performed. Data show means ± SD based on one-way ANOVA. *** *P* < 0.001.

To further examine the relationship between BMAL1, *Mex3a* and *Lgr5* expression in intestinal cells, *Bmal1*-depleted mIEC cells were subjected to immunoblotting assays. A decrease in MEX3A and LGR5 protein level was observed upon *Bmal1* knockdown (Fig. 3A and B). Consistent with the observation that *Mex3a* knockout decreased LGR5^+^ CBCs^11^, we also found that depletion of *Mex3a* with shRNA decreased LGR5 protein levels (Fig. 3A and C). Also, overexpression of either BMAL1 in *Mex3a*-depleted cells or MEX3A in *Bmal1*-depleted cells (using pBMAL1-His or pMEX3A-Flag plasmids) partially recovered the reduction of LGR5 caused by *Bmal1* or *Mex3a* knockdown (Fig. 3D). Together, these data indicated that both BMAL1 and MEX3A contribute to LGR5 expression and that BMAL1 regulates LGR5 expression via at least two pathways. In one of these pathways, BMAL1 acts via *Mex3a* expression while in the other BMAL1 may directly regulates *Lgr5* transcription (Fig. 3E).

### BMAL1 directly upregulates *Mex3a* transcription

**Figure 4:**
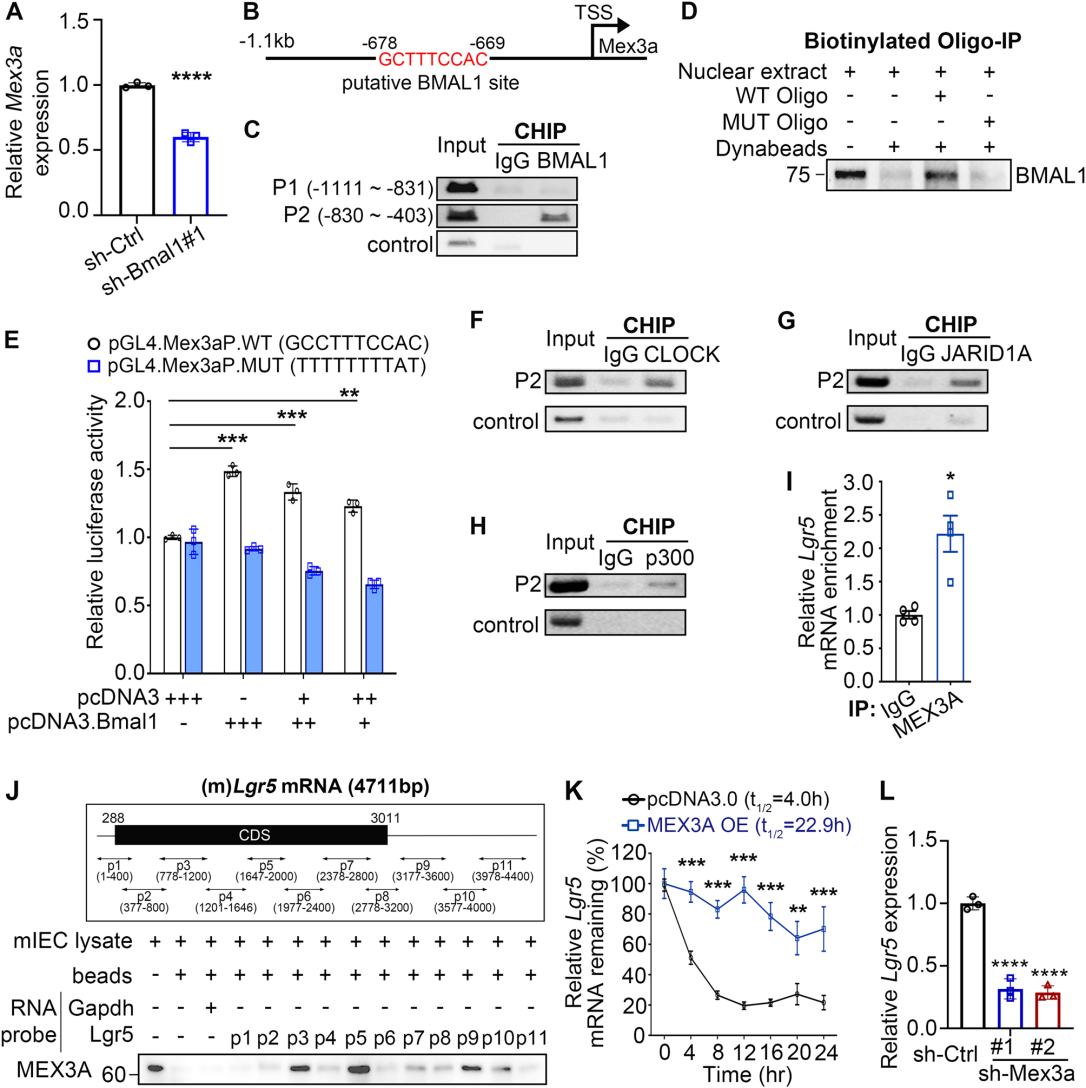
BMAL1 transcriptionally upregulates *Mex3a*. (**A**) qRT-PCR analysis of *Mex3a* in sh-Ctrl or sh-Bmal1 mIEC cells. *GusB* was used as an internal control. Three independent experiments were performed and data are means ± SD from one representative experiment. Significant differences are based on unpaired T-test. (n = 3, ****P < 0.0001). (**B**) Diagram shows one predicted BMAL1 binding site on the mouse *Mex3a* promoter using the JASPAR database. (**C**) ChIP analysis of BMAL1 occupancy on the *Mex3a* promoter in mIEC cells. Mouse IgG and far site control region (FSC; Chr 3, 88,521,891–88,522,294) were used as negative controls. Gel images of BMAL1 occupancy on P1 and P2 regions are from one representative experiment of two independent experiment. The original gel images and additional experiments with similar results are shown in the “Supplementary information” file. (**D**) Biotinylated oligo pull-down of BMAL1 using WT or mutant predicted BMAL1 binding sequence and mIEC nuclear extract. Blots shown are from one representative experiment of three independent experiments. The original blots and additional experiments with similar results are shown in the “Supplementary information” file. (**E**) Top: Schematic diagram of pGL4.10-Mex3a promoter-Luc reporter with WT BMAL1 binding sequences. Bottom: Luciferase reporter assays were conducted using 293T cells co-transfected with pcDNA3.0-BMAL1 and the WT (black bars) or mutant (MUT, blue bars) *Mex3a* promoter constructs. Three replicate experiments were performed. Data show means ± SD based on two-way ANOVA. ** *P* < 0.01, *** *P* < 0.001. (**F**–**H**) ChIP analysis of CLOCK (**F**), JARID1A (**G**) and p300 (**H**) on the Mex3a promoter using nuclear extract from mIEC cells. IgG and far site region (FSC) were used as an IP control. Gel images of CLOCK, JARID1A and p300 occupancy on P2 region are from one representative experiment of two independent experiments. The original gel images and additional experiments with similar results are shown in the “Supplementary information” file. (**I**) RNA-IP assay of MEX3A-*Lgr5* complex using mIEC lysates. IgG was used as an antibody control for IP. Three independent experiments were performed. Data are presented as means ± SD, significant differences are based on Student’s T test. * *P* < 0.05. (**J**) Top: Schematic of mouse *Lgr5* mRNA and 11 biotinylated RNA probes (P1–P11) spanning nearly whole *Lgr5* mRNA sequence used for RNA pull-down assay. Bottom: RNA pull-down assay of MEX3A using 11 biotinylated RNA probes that covered the Lgr5 coding regions (CDS) and 3′-UTR. Beads only and the Gapdh RNA probe were used as pull-down controls. Blot shown is from one representative experiment of three independent experiments. The original blots and additional experiments with similar results are shown in the “Supplementary information” file. (**K**) 5-bromouridine (BrU) IP chase assay (BRIC) of *Lgr5* mRNA in mIEC cells transfected with pcDNA3.0 control vector or pMex3a-3X flag (MEX3A OE). *Lgr5* levels at 0 h was set as 100%. Two independent experiments were performed. Data show means ± SD, significant differences are based on two-way ANOVA. ** *P* < 0.01, *** *P* < 0.001. (L) qRT-PCR analysis of *Lgr5* levels in mIEC transduced with sh-Ctrl or sh-Mex3a lentiviruses. Three independent experiments were performed. Data show means ± SD, significant differences are based on one-way ANOVA. ** *P* < 0.01, *** *P* < 0.001.

Given the close relationship between *Bmal1* depletion and MEX3A protein and mRNA downregulation (Figs. 3A, 4A), we analyzed the *Mex3a* promoter using the JASPAR database^32^ and found a putative BMAL1 binding site [GCTTTCCAC; -678~-669 nt upstream of the transcriptional start site (TSS)] (Fig. 4B). Even though this site is not a complete match to the BMAL1 consensus binding site [E-box (E1, CACGTG) or E1-E2 (E2, AACGTG) tandem sites]^33^, chromatin-immunoprecipitation (ChIP) assay using mIEC cells found BMAL1 associated with this *Mex3a* promoter region (Fig. 4C). Biotinylated-oligonucleotide-pull-down assay demonstrated that BMAL1 directly and specifically bound to this site, as BMAL1 bound an oligo probe containing this putative site but not a mutated probe (Fig. 4D). Transient reporter assay showed that mutation of this site abolished BMAL1 transactivation of the *Mex3a* promoter in HEK-293T cells (Fig. 4E). Consistent with previous reports that BMAL1-CLOCK heterodimers^19^ enhance their transcriptional activity by interacting with chromatin-modifying proteins such as JARID1A^34^ and CBP/p300^35^, BMAL1 interacted with these factors in mIEC cells (Fig. S1). The BMAL1-CLOCK-JARID1A-p300 complex was on the *Mex3a* promoter to activate *Mex3a* transcription (Fig. 4F–H). Together, these results indicated that BMAL1 directly activates *Mex3a* expression.

### MEX3A binding stabilizes *Lgr5* mRNA

Even in BMAL1 expressing mIEC cells, *Mex3a* depletion still reduced LGR5 level (Fig. 2C), suggesting that MEX3A can affect LGR5 levels independently of BMAL1. RNA immunoprecipitation (RNA-IP) assay showed that MEX3A bound to *Lgr5* mRNA (Fig. 4I). This suggested that MEX3A may regulate *Lgr5* expression post-transcriptionally. Since the RNA motif and secondary structure recognized by MEX3A is not known, we used eleven biotinylated-RNA probes (400–500-nt in length per probe) spanning the *Lgr5* mRNA including the 3′-UTR (Fig. 4J) to perform biotinylated-RNA pull-down assay. The results showed that MEX3A interacted with *Lgr5* mRNA at multiple sites, including two in the coding region (CDS, probes 3, 5) and one in the 3′-UTR (probe 9) (Fig. 4J). MEX3A binding stabilized *Lgr5* mRNA as MEX3A overexpression significantly increased the half-life of *Lgr5* mRNA from 4 to 22.9 h in mIEC (Fig. 4K). Consistent with these observations, depletion of MEX3A reduced *Lgr5* mRNA level in mIEC cells (Fig. 4L). Together, these data indicated that BMAL1 could directly activate *Lgr5* transcription and indirectly stabilize *Lgr5* mRNA by upregulating MEX3A.

## Discussion

**Figure 5:**
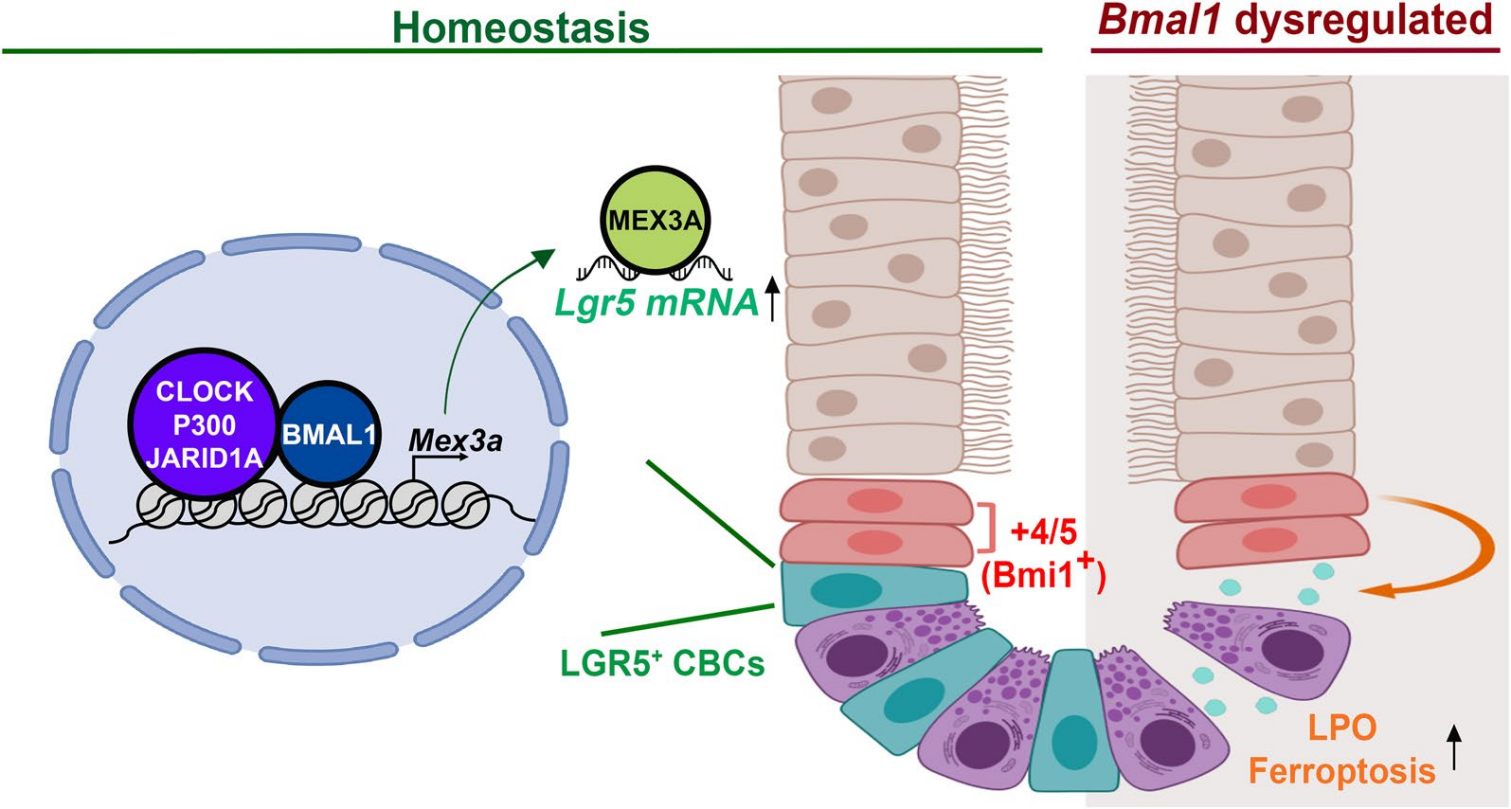
Model of BMAL1 function in maintaining intestinal homeostasis. In intestinal crypts, BMAL1 is crucial to maintain homeostasis between the fast-proliferating LGR5^+^ CBCs and damage-resistant BMI1^+^  +4 cells. BMAL1, together with CLOCK, JARID1A and p300, upregulated expression of *Mex3a*; while MEX3A further enhanced LGR5 expression by directly binding and stabilizing *Lgr5* mRNA. Ablation of *Bmal1* not only reduced both *Mex3a* and *Lgr5* expression, but also increased lipid peroxidation (LPO) and ferroptosis, resulting in decreased LGR5^+^ CBC prevalence but increased BMI1 expressing cells in crypts.

The circadian clock modulates several aspects of intestinal physiology including IEC regeneration^36^, microbiota-IEC crosstalk^37^, and intestinal permeability^38^. BMAL1 is essential for maintaining rhythmic cell division within intestinal organoids^17^ and for coordinating intestinal regeneration^16^. Despite some controversy of whether LGR5^+^ CBCs are dispensable in intestinal tissue regeneration and tissue homeostasis^7^, convincing evidence has shown that LGR5^+^ CBCs are required to maintain a healthy intestinal epithelium^5^. Our results show that BMAL1 is essential for *Lgr5* expression in the crypt cells and therefore may contribute to maintaining intestinal stem cell homeostasis. Using two *Bmal1* knockout mouse models, we showed that ablation of *Bmal1* reduced *Mex3a* and *Lgr5* expression as well as LGR5^+^ CBC numbers but increased BMI1^+^ cells in the crypts (Fig. 5). We further demonstrated that BMAL1 increased MEX3A to stabilize *Lgr5* mRNA in mIEC cell line. Our results, together with the known role of MEX3A in maintaining the LGR5^+^ CBC pool^11^, suggest a possible role of BMAL1 in regulating intestinal stem cell succession, and thereby contributing to intestinal homeostasis. Intriguingly, we did not observe growth deficiency in BMAL1 knockout intestinal epithelium despite the significant loss of LGR5^+^ CBCs (Fig. 1). This phenotypic observation is consistent with the previous report that BMAL1 knockout mice had no obvious difference in tissue growth but did have reduced numbers of intestinal LGR5^+^ CBC and crypts^16^. Interestingly, we observed some LGR5/GFP^-^ crypt cells expressing BMAL1 in the *LC*^+^*B*^*fl/fl*^ intestine (Fig. 1B). A possible explanation is that BMI1^+^  +4 cells turned on BMAL1 expression to facilitate reprogramming and thus replenish the loss of LGR5^+^ CBCs in the crypts. However, the mosaicism in the *Lgr5-Cre* mouse model in which GFP expression does not reflect the actual LGR5 expression level in the crypts^39^ makes it difficult to determine whether BMAL1 detected in the crypt cells was due to BMI1^+^  +4 cells which underwent reprograming to replenish the loss of LGR5^+^ cells or due to incomplete knockout in the LGR5^+^ CBCs. A careful lineage tracing of LGR5^+^ CBCs and BMI1^+^  +4 cells after *Bmal1* is conditionally knocked out, either in vivo or using enteroids, will be required to more conclusively investigate this point and to further evaluate if BMAL1 and MEX3A promote BMI1^+^  +4 cell reprogramming to LGR5-expressing cells. Nevertheless, our mechanistic findings have multiple implications for how circadian time may affect intestinal damage and recovery in response to various stimuli.

In addition to regulating *Mex3a* and *Lgr5* expression, BMAL1 may also contribute to crypt homeostasis by protecting crypt cells from lipid peroxidation and ferroptosis. Several lines of evidence have shown that BMAL1 dysregulation results in ferroptosis; however, the underlying mechanisms vary among different cell types. For example, in fibrosarcoma and non-small-cell lung cancer cell lines, autophagy-mediated BMAL1 degradation facilitates EGLN2 expression to destabilize HIF1A which ultimately increases lipid peroxidation^40^. In pancreas, conditional knockout of *Bmal1* inhibits antioxidant defense genes such as SLC7A11 (an antiporter mediating the uptake of extracellular cystine in exchange for glutamate), GPX4 and superoxide dismutase 1 (SOD1) and consequently increases ferroptosis-associated pancreatitis^41^. Consistent with these reports, we also found that *Bmal1* depletion in mIEC cells significantly elevated lipid peroxidation and intracellular Fe^2+^ levels (Fig. 2C and D), likely via downregulation of GPX4 (Fig. 2E). In a parallel manner, *Mex3a* knockdown also led to GPX4 suppression and ferroptosis elevation (Fig. 2E). Whether BMAL1 and MEX3A directly regulate GPX4 or other targets in ferroptosis pathways remain to be elucidated. Interestingly, *Mex3a* deficiency has been shown to upregulate the peroxisome proliferator‐activated receptor γ (PPARγ) pathway^11^. The PPAR family, including PPARα, PPARδ and PPARγ, have all been suggested to decrease ferroptosis^42–44^. As ferroptosis is regulated by several pathways^45^, it is unclear how GPX4, the primary enzyme that prevents ferroptosis^31^, and PPAR or other lipid peroxidation regulatory pathways interact in the intestinal crypt cells under *Bmal1* depleted conditions. This topic requires further investigation. Moreover, depletion of *Bmal1* or *Mex3a* can result in apoptosis^46,47^ or alter tissue inflammatory status^48^ which consequently may lead to pyroptosis^49^. Whether BMAL1 also contributes to crypt homeostasis by preventing these cell death pathways will also be of interest for further investigation.

It should also be noted that the role of BMAL1 in colonic homeostasis has been described using intestinal tissue specific *Bmal1* knockout and *Apc*^min/+^ mouse models^50,51^. BMAL1 is critical for colonic epithelial regeneration which contributes to colitis recovery^50^. In *Apc*^+/+^ and *Apc*^min/+^ mice lacking *Bmal1*, BMAL1 was found to regulate intestinal stem cell pathways, such as Hippo signaling^51^. Loss of *Bmal1* perturbed the homeostasis of stem cell population and increased tumor initiation^51^. However, to our knowledge, the regulatory relationship between BMAL1, MEX3A and *Lgr5* has not been described in the colon. Whether the BMAL1-MEX3A-*Lgr5* regulatory mechanism identified in duodenal crypts is also applicable in colonic crypts remains to be investigated. Intriguingly, colorectal cancer stem cells have high *Lgr5* expression^52^. Whether these cancer stem cells are able to upregulate *Lgr5* expression in a BMAL1 independent manner and how BMAL1, MEX3A and *Lgr5* regulatory relationship in colorectal cancer stem cells differs from that in non-cancerous intestinal tissue will also be of interest for further investigation.

Consistent with previous observations that MEX3A is essential for the survival of LGR5^+^ cells and organoid forming abilities^11^, our study further demonstrated that MEX3A is transcriptionally upregulated by BMAL1 (Fig. 4) and plays an essential role to co-regulate ISC homeostasis with BMAL1 (Figs. 1 and 2). Moreover, LGR5^+^ CBCs can be divided into two subpopulations with different proliferation rate and stress response based on MEX3A expression level^15^. The MEX3A^high^-LGR5^+^ cells are more stress resistant and critical for intestinal epithelium repair and homeostasis upon toxic insults^15^. Since BMAL1 drives rhythmic gene expression and regulates biological functions under circadian control^53^, it is possible that the expression of MEX3A, and therefore the prevalence of the MEX3A^high^-LGR5^+^ cells, also fluctuate with BMAL1 oscillation through the day-night cycle. Interestingly, in C57BL/6J mice, BMAL1 protein oscillated in a period shorter than 24 h in the duodenum crypt cells and *Mex3a* expression oscillated with a pattern that coincided with BMAL1 expression [*p*-value 0.038 using cosinor analysis (https://cosinor.online, period length = 12 h)] (Fig. S2). However, *Lgr5* mRNA did not have a statistically significant oscillation (*p* = 0.1, period length = 12 h). How and why the crypt cells exhibited a shorter period of BMAL1 oscillation remains to be determined. Recently, a few studies have used enteroid culture and colon tumor organoids from transgenic mice to examine circadian rhythms and BMAL1 regulated genes. In these published RNA-seq data, *Mex3a* rhythmic expression was not detected^51,54^. Enteroid culture is currently the best in vitro model to mimic intestinal epithelium. However, each enteroid contains multiple epithelial cell types and the proportion of stem cells is low. Thus, the oscillation of *Mex3a* transcript level in stem cells is likely to have been masked due to the high stoichiometry of other cell types. As for colon tumor organoids, they have no self-autonomous clock function^51^ and thus no rhythmic *Mex3a* was detected. Therefore, techniques such as single cell RNA sequencing using intestinal crypts at different times of day will be required to evaluate whether *Mex3a* and *Lgr5* oscillate in CBCs. Nevertheless, consistent with our mechanistic findings, tumor organoids derived from an *Apc*^min/−^ mouse model lacking Bmal1 (*Apc*^min/−^; Bmal1^−/−^) found that both *Lgr5* and *Mex3a* were significantly downregulated compared to organoids derived from *Apc*^min/−^; Bmal^+/+^ tumors^51^ indicating that *Lgr5* and *Mex3a* are regulated by BMAL1.

In metastatic colon cancer patients, several random trials have tested time-modulated combination treatment of 5-FU, oxaliplatin and leucovorin/folinic acid and found that drug delivery between 2 and 4 o’clock in the morning, instead of constant infusion, showed higher tumor objective response rates (> 50%)^55,56^ and milder mucositis^56^. The main goal of such chrono-therapeutic strategy is to deliver chemotherapeutic agents at specific circadian time points to maximize drug efficacy and minimize adverse effects^57^. Our results indicate that drug delivery at the time of maximal BMAL1 and MEX3A expression in intestinal stem cells may allow intestinal side-effects of drugs such as 5-FU to be minimized. More detailed mechanistic studies are needed to determine whether this BMAL1-MEX3A-*Lgr5* regulation is circadian dependent which may provide insights into the mechanisms of chrono-therapeutic response and could facilitate development of more effective treatment strategies for intestinal cancer and other diseases.

## Materials and methods

### Mouse breeding and maintenance

All animal breeding and methods were carried out in accordance with relevant guidelines and regulations, and were approved by the Academia Sinica Institutional Animal Care and Utilization Committee (AS IACUC# 17-12-1165). All mice were maintained under standard 12:12 h light/dark (LD) condition, ZT0 (7:00 AM) corresponded to lights-on and ZT12 (7:00 PM) corresponded to lights-off. To reduce animal use to fulfill the 3Rs (Replacement, Reduction and Refinement) principle, we examined BMAL1 protein level and observed a 24-h oscillation in the duodenum tissue (containing both crypt cells and differentiated epithelial cells in the villus) of C57BL/6J mice kept under LD condition (Fig. S3). BMAL1 protein expression was high during the light phase (peak at ZT5), but low during the dark period in the duodenum epithelium. We therefore decided to choose ZT5 for our animal experiments. To specifically knockout *Bmal1* expression in LGR5^+^ CBCs in the intestine, *Lgr5-EGFP-ires-CreERT2* (referred as *Lgr5-Cre*; JAX stock no. 008875) mice^2^ were crossed with *Bmal1* floxed mice with two *loxP* sites flanking exon8 of mouse *Bmal1* (*Bmal1*^*fl/fl*^, JAX stock no. 007668)^58^. To improve animal welfare, repetitive intraperitoneal administration or gavage which can cause stress, injury or local inflammation to the mice^59,60^ was avoided. The progeny (*Lgr5-Cre*^+^*; Bmal1*^*fl/fl*^*, Lgr5-Cre*^+^*; Bmal1*^*wt/wt*^) were fed with tamoxifen (TAM) diets (ENVIGO, Cat#TD130856) for 3 weeks to activate the CreERT2 recombinase (Fig. 1A). To knockout *Bmal1* expression in intestinal epithelial cells, *B6.Cg-Tg(Vil1-cre)1000Gum/J* (referred as *Vil-Cre*, JAX stock no. 021504) mice^61^ were crossed with *Bmal1*^*fl/fl*^ mice to generate *Vil-Cre*^+^*;Bmal1*^*fl/fl*^ (*Vil-Cre*^+^*B*^*fl/fl*^) mice. All experiments were performed using duodenum (approximately 1 cm distal to pyloric sphincter) of male mice at 8–12 weeks of age. Euthanasia was performed by carbon dioxide inhalation. All methods are reported in accordance with ARRIVE guidelines (https://arriveguidelines.org).

### Cell lines

Small intestinal epithelial cell line (mIEC) from mouse E19.5 fetuses [^62^, Insreenex Cellular Screening Technologies, Cat# INS-CI-1007] was maintained in muINTEPI medium (Cat# INS-ME-1005) with basal supplement (Cat# ME1005BS) in Type I collagen coated plates. Cells were cultured at 37 °C in a 5% CO_2_ humidified incubator.

### Plasmids and reagents

The lentiviral pLKO-puro-shRNA expression vectors sh-Ctrl (TRC005), sh-Bmal1#1 (TRCN282303), sh-Bmal1#2 (TRCN282305), sh-Mex3a#1 (TRCN255045) and sh-Mex3a#2 (TRCN255048) were from the National RNAi Core Facility (Academia Sinica).

pcDNA3.Bmal1-His was made by inserting Bmal1-His cDNA from pBMPC3 (pBmal1-His) (Addgene, Cat# 31367). pMex3a-3X Flag was from GeneCopoeiaTM (Cat# EX-Mm32128-M14). All constructs were verified by DNA sequencing.

### Immunoblot assay

Whole cell lysate was prepared using RIPA buffer (150 mM NaCl, 1% NP-40, 0.5% sodium deoxycholate, 0.1% SDS, 50 mM Tris pH8.0, SIGMAFAST™ Protease Inhibitors (Sigma-Aldrich, Cat# S8830-20TAB) and PhosSTOP EASYpack phosphatase inhibitor cocktail (Roche, Cat# 4906837001). Protein concentration was determined by Bradford assay (Bio-Rad, Cat# 5000006). 50–30 μg total proteins were separated by 7.5% or 10% tris–glycine polyacrylamide gel, with overnight incubation with primary antibodies (Table S1) and followed by a 1:10,000 dilution of horseradish peroxidase (HRP)-conjugated secondary antibodies. HRP signals were detected using Western Lightning® Ultra Chemiluminescence Substrate (PerkinElmer INC., Cat# NEL113001EA) and images captured by a UVP ChemStudio Plus BioImaging system. The densitometry of blot bands was quantified using Image Lab 6.0 software.

### Immunohistochemical (IHC) and Immunofluorescence (IF) staining

Tissues were fixed in 10% formalin overnight at 4 °C and embedded in paraffin. Sections were cut into 4 μm slices, dewaxed with xylene and rehydrated with descending ethanol series to water. Antigen retrieval was performed using the citrate-based antigen unmasking solution (Target Retrieval Solution (10X), Dako, Cat# S1699) for 20 min under high pressure condition. Slices were stained with primary antibodies (Table S1) overnight at 4 °C, followed by incubation with DAKO REAL™ EnVision™ HRP Labeled Polymer Anti-Rabbit (Cat# K4003) at room-temperature for 30 min. The 3.3′-diamiobenzidine (DAB) substrate was used to detect the peroxidase activity, and the slices were counterstained with hematoxylin. Slides were photographed under 40× magnification by the Aperio scanner machine (Leica Biosystems, Singapore). For IF staining, tissues were hybridized with mouse anti-BMAL1 (1:50), rabbit anti-MEX3A (1:50) or chicken anti-GFP (1:500) primary antibodies overnight at 4 °C after de-paraffined, rehydration and antigen retrieval. After washed by PBST three times, the slices were incubated with anti-mouse (1:1000), anti-rabbit polymer-HRP (1:1000) or anti-chicken CF488A (1:200) secondary antibodies at room-temperature for 1 h. To enhance the fluorescence signal, the anti-BMAL1 and anti-MEX3A hybridized slides were further labeled with TSA™ plus cyanine 3 (1:1500, PerkinElmer INC., Cat# NEL744B001KT) or TSA™ plus cyanine 5 (1:1500, Cat# NEL745B001KT), respectively. Images were obtained by a laser scanning confocal microscope (LSM770, Carl Zeiss MicroImaging). The fluorescence intensity was quantified using the QuPath software.

### RNAscope in situ hybridization

*Lgr5*, *Mex3a* and *Bmi1* mRNA expression were detected using either RNAScope® Multiplex Fluorescence Detection Reagent V2 kit (Advanced Cell Diagnostics, Cat# 323110) or RNAScope® 2.5 HD Detection Reagent-Brown kit (Cat# 322310) with Mm-Lgr5 (Cat# 312171), Mm-Mex3a-E2-CDS (Cat No. 318551) and Mm-Bmi1-O1 (Cat No. 466021) probes according to the manufacturer’s instructions. Quantification of mRNA positive spots were performed using the trainable Weka segmentation classifier in Fiji software according to the recommendation of TECHNICAL NOTE from Advanced Cell Diagnostics.

### Flow cytometry (FACS) for lipid peroxidation analysis

For lipid peroxidation detection, cells were stained with 0.75 μM BODIPY 581/591 C11 (Thermo Fisher Scientific, D3861) for 30 min at 37 °C. Oxidation of the polyunsaturated butadienyl structure in BODIPY-C11 was measured by fluorescence emission at 600 nm with excitation at 570 nm. FACS analysis was performed using a FACS Canto II system (BD Biosciences).

### FerroOrange staining

One million cells were seeded in a 35 mm plate and cultured overnight before subjected to FerroOrange staining according to the manufacturer’s instructions. In brief, cells were washed with HBSS (Gibco, Cat# 14175-095) three times and then stained with 1 µmol/L FerroOrange (Dojindo, Cat# F374) in serum free medium for 15 min at 37 °C. Images were acquired using an Andor Dragonfly 202 high speed confocal microscope system and the level of labile iron (II) ions (Fe2^+^) was measured by fluorescence emission at 572–615 nm with excitation at 561 nm. The fluorescence intensity was quantified using the Imaris 9.9 software.

### Quantitative real-time PCR (qRT-PCR)

RNA was extracted using TRI Reagent (Sigma-Aldrich, Cat# T9424) according to the manufacturer’s instructions. For mRNA expression detection, 3 µg total RNA from each sample was reverse-transcribed using RevertAid First Strand cDNA Synthesis Kit (Thermo Fisher Scientific, Cat# K1622). 10 ng 1st-strand cDNA was used for real-time PCR with appropriate primer sets and SYBR® Green PCR Master Mix (Thermo Fisher Scientific, Cat# 4309155) using ABI-7900 thermocycler. The mRNA relative quantities were determined using comparative cycle threshold methods with Beta-glucuonidase (GusB) as an internal control. Primers for qRT-PCR were 5′- GCAGGCAAGGCTGCAAGATT -3′ (forward) and 5′- ACTTGTTGCGTGAGGCTCTT -3′ (reverse) for *Mex3a*, 5′- CCTTCCCCAGGTCCCTTCAA -3′ (forward) and 5′-GAACACGGTCAAAGCCACCA-3′ (reverse) for *Lgr5*, and 5′- CCGACCTCTCGAACAACCG -3′ (forward) and 5′- GCTTCCCGTTCATACCACACC -3′ (reverse) for *GusB*.

### Chromatin immunoprecipitation (ChIP) assay

Cells were crosslinked with 1% formaldehyde (Sigma-Aldrich, Cat# F8775) for 10 min at 37 °C and the crosslinking was stopped by 0.125 M glycine. After PBS wash, the crosslinked cells were lysed in 1 mL cell lysis buffer (10 mM Tris–HCL pH8.0, 1 mM EDTA, 0.5% NP-40, 1 mM PMSF, SIGMAFAST™ protease inhibitor cocktail, and Roche PhosStop) on ice for 20 min. Nuclei pellet was then resuspended and incubated in 1 mL of nuclear lysis buffer (50 mM Tris–HCL pH8.0, 100 mM EDTA, 1% SDS, 1 mM PMSF, SIGMAFAST™ protease inhibitor cocktail, and Roche PhosStop) on ice for 30 min and 3 freeze–thaw cycles to release chromatin. After centrifugation, the crosslinked chromatin was resuspended in 80 µL nuclear lysis buffers and sonicated using a Diagenode Bioruptor®pico (15 cycles of 30 s on and 30 s off) to 200–500 bases. A 10 µL aliquot of the fragmented chromatin was used as an input control. For immunoprecipitation assay, pre-washed protein A/G magnetic beads (Thermo Fisher Scientific, Cat# 88803) were incubated with 4 µg anti-BMAL1, 9 µg anti-CLOCK, 9 µg anti-p300, 6 µg anti-JARID1A, or control IgG antibody (Table S1) at 4 °C overnight. 25 µg sonicated chromatin was diluted to a final volume of 1 mL with dilution buffer (20 mM Tris–HCL pH 8.0, 150 mM NaCl, 1 mM EDTA, 0.01% SDS, 1% Triton X-100), and pre-cleaned with pre-washed protein A/G magnetic beads and 10 µg mouse-IgG isotype control at 4 °C overnight. The antibody-coupled beads were then incubated with the sonicated chromatin for 4 h at 4 °C. After washed several times, the chromatin-protein complex was reversed crosslinked by addition of 15 µL solution containing 2.5 M NaCl, 2 µL 50 mg/mL RNaseA and 6 µL 10 mg/mL proteinase K. DNA was extracted by MinElute® PCR purification kit (Qiagen, Cat# 28004). Semi-quantitative PCR was performed to detect BMAL1 associated promoter regions using primers listed in Table S2.

### Luciferase reporter assay

Mus muscles strain *C57BL/6JNarl* chromosome 3 (88,530,849–88,533,098 bp) containing the proximal promoter of *Mex3a*, *Mex3a* exon1 and part of the coding sequence, was amplified by PCR from mIEC genomic DNA and cloned into pGL4.10 vector (Promega, Cat#E6651) (referred as pGL4.Mex3aP.WT). The putative BMAL1 binding site predicted by the JASPAR database was mutated from 5′-GCCTTTCCAC-3′ to 5′-TTTTTTTTAT-3′ (referred as pGL4.Mex3aP.MUT). pGL4.74 Renilla luciferase plasmid (pGL-4.74[hRluc/TK], Promega, Cat# E692A) was used as a transfection control. For HEK-293T cells, 6 × 10^5^ cells were seeded and co-transfected with 500 ng of pGL4.Mex3aP.WT or pGL4.Mex3aP.MUT, 6.25 ng of pGL-4.74[hRluc/TK], pcDNA3 or pcDNA3.Bmal1-His (range from 300 to 600 ng) using TransIT-LT1 transfection reagents (Mirus Bio LLC., Cat# MIR2300). Cell extracts were collected 48 h after transfection and the luciferase activity was measured using Dual-Luciferase® Reporter (DLR™) Assay System (Promega, Cat# E1910).

### Co-immunoprecipitation (Co-IP) assay

Nuclear extraction was done using Subcellular Protein Fractionation Kit (Thermo Fisher Scientific, Cat# 78840) according to the manufacturer's instruction. For IP, 300 µg nuclear extract was incubated with 4 µg anti-BMAL1, 9 µg anti-CLOCK, 9 µg anti-p300, 6 µg anti-JARID1A, or control IgG antibody at 4 ℃ overnight. The antibody-protein complexes were precipitated using 20 µL pre-washed and pre-blocked (10% BSA) protein A/G agarose beads (Thermo Fisher Scientific, Cat# 20422) at 4 ℃ for 1 h. After washed with NP-40 TNE buffer (10 mM Tris–HCl pH7.5, 150 mM NaCl, 0.5 mM EDTA, 0.1% NP-40, and protease inhibitor cocktail) five times, interacting proteins were eluted with SDS-PAGE and detected by western blotting assay.

### RNA immunoprecipitation (RIP) assay

Whole-cell lysate from 2 × 10^7^ mIEC was used for RIP assay using Magna RIP™ RNA-Binding Protein Immunoprecipitation Kit (Millipore, Cat# 17-700) according to the manufacturer’s instruction. 4 µg anti-MEX3A antibody was used for the immunoprecipitation at 4 ℃ overnight, and rabbit IgG was used as a pull-down control. MEX3A bound RNAs were then isolated following the procedures of proteinase K digestion and phenol–chloroform-isoamyl alcohol extraction. The precipitated RNAs were resuspended in 12 µL DEPC-ddH_2_O. Quantitative PCR was performed to determine the level of MEX3A-bound *Lgr5* mRNA.

### RNA pull-down assay

Biotin-14-CTP (Thermo Fisher Scientific, Cat# 19519016) labeled RNA was in vitro transcribed using MEGAshortscript™ T7 High Yield Transcription Kit (Thermo Fisher Scientific, Cat# AM1354) according to the manufacturer's instruction. After purification with RNeasy Plus Mini Kit (Qiagen, Cat# 74136), 2 µg biotinylated RNAs was incubated with 20 µL pre-washed Dynabeads™ M-270 Streptavidin (Thermo Fisher Scientific, Cat# 65305) in 1 mL buffer (20 mM Tris–HCl (pH7.5), 100 mM KCl, 5 mM MgCl_2_, and 0.5% NP-40), and incubated at 4 °C for 1 h with gentle agitation. 450 µg mIEC cytoplasmic protein lysate was added to the washed RNA-probe coupled dynabeads, and incubated in 1 × TENT buffer (10 mM Tris–HCl (pH8.0), 1 mM EDTA (pH 8.0), 250 mM NaCl, and 0.5% Triton X-100) with SIGMAFAST™ protease inhibitor cocktails and RNaseOUT™ Recombinant Ribonuclease Inhibitor (Thermo Fisher Scientific, Cat# 10777019) at 4 °C for 8 h. After washed with 1 × TENT buffer, the proteins interacted with mouse *lgr5* probes were eluted using 40 µL SDS buffer and detected by immunoblot assay. Biotinylated *Gapdh* RNA was used as a pull-down control. All RNA-probes are listed in Table S3.

### BRIC assay

mIEC was transfected with pcDNA3.0 or pMex3a-3X Flag and then incubated in the 5′-bromo-uridine (BrU, Sigma-Aldrich, Cat# 850187) containing medium to a final concentration of 1 mM in a humidified incubator with 5% CO_2_ for 8 h. After removal of BrU, RNA was harvested at indicated time points using the TRI Reagent®. 15 µL pre-washed Dynabeads™ Protein G (Thermo Fisher Scientific, Cat# 10004D) was blocked with 2% BSA and 5 mg heparin (Santa Cruz, Cat# 203075) at 4 °C for 1 h, and then incubated with 4 µg anti-BrdU Ab at 4 °C for 1 h. The BrdU Ab coupled dynabeads were further washed using BrU-IP buffer and 250 mM NaCl three times and resuspended in 15 µL BrU-IP buffer supplemented with RNaseOUT™ Recombinant Ribonuclease Inhibitor. To normalize the immunoprecipitation of BrU-labeled RNAs among different time points, the in vitro transcribed BrU-labeled luciferase RNA was used as a spike-in control for internal standard of quantitative PCR assay. 50 μg BrU-labeled RNAs from mIEC and 100 ng BrU-labeled luciferase RNA were mixed in 1 mL BrU-IP buffer with RNaseOUT, and the RNA mixture was denatured by heating at 80 °C for 3 min. Subsequently, 15 µL BrdU Ab conjugated dynabeads were added into RNA mixture and incubated at room temperature for 1 h. After washed four times with BrU-IP buffer (20 mM Tris–HCL pH7.5, 250 mM NaCl, and 20 μL RNaseOUT), the BrU-labeled RNAs were isolated using RNeasy Plus Mini Kit. The half-life of *lgr5* mRNA was determined by RT-qPCR assay.

### Statistical analysis

Quantitative results were represented as Mean ± SD. Statistical analysis was conducted using the Graph Pad Prism 9.0 (GraphPad Software). Non-parametric Mann–Whitney test was used to compare control and experimental genotypes or treatment groups. Statistical significance among more than three groups was analyzed using one-way ANOVA with Dunn’s multiple comparison test. For the BRIC assay, two-way ANOVA with Sidak multiple comparison test was performed to detect significance among different groups. Asterisk (*, **, ***) indicates statistical significance with *p*-value < 0.05, 0.01, 0.001, respectively.

### Supplementary Information

Supplementary Information.

## Data Availability

All data are available in the main text or the supplementary materials. Requests for further information, reagents, and resources should be directed to and will be fulfilled by the corresponding author, Wendy W. Hwang-Verslues (wendyhv@gate.sinica.edu.tw).
