# Supplementary material for: Core clock gene BMAL1 and RNA-binding protein MEX3A collaboratively regulate Lgr5 expression in intestinal crypt cells
Source: Sci Rep. 2023 Oct 16;13:17597. doi: 10.1038/s41598-023-44997-5 (PMC10579233; doi:10.1038/s41598-023-44997-5)

Supplementary material

Core clock gene BMAL1 and RNA-binding protein MEX3A collaboratively regulate Lgr5 expression in intestinal crypt cells

Li-Tzu Cheng, Grace Y.T. Tan, Fang-Pei Chang, Cheng-Kai Wang, Yu-Chi Chou, Pang-Hung Hsu, Wendy W. Hwang-Verslues*

Corresponding Author: Wendy W. Hwang-Verslues
Email: wendyhv@gate.sinica.edu.tw

This PDF file includes:

Fig. S1. BMAL1 interacted with CLOCK, JARID1A and p300 in mIEC cells.
Fig. S2. Oscillation of BMAL1 protein, *Mex3a* and *Lgr5* mRNA in duodenal crypts of C57BL/6J mice.
Fig. S3. Oscillation of BMAL1 protein in duodenum of C57BL/6J mice.
Table S1. Antibodies.
Table S2. Primer sequence for ChIP analysis.
Table S3. Probe sequence for RNA pull-down assays.

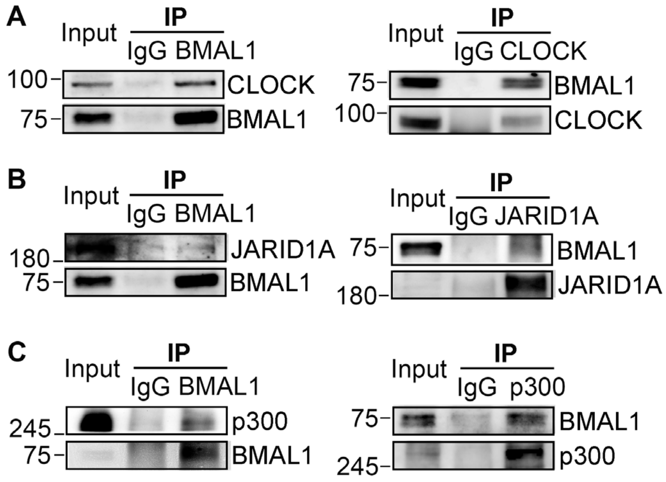

Fig. S1. BMAL1 interacted with CLOCK, JARID1A and p300 in mIEC cells.

Co-IP and reciprocal-IP analysis of BMAL1 and CLOCK (**A**), JARID1A (**B**) or p300 (**C**) using nuclear extract from mIEC cells. IgG was used as an IP control. Blots shown are from one representative experiment of at least two independent experiments. Images of BMAL1 in (A) and (B) are from the same blot. The original blots and additional experiments with similar results are shown in this "Supplementary information" file (see below).

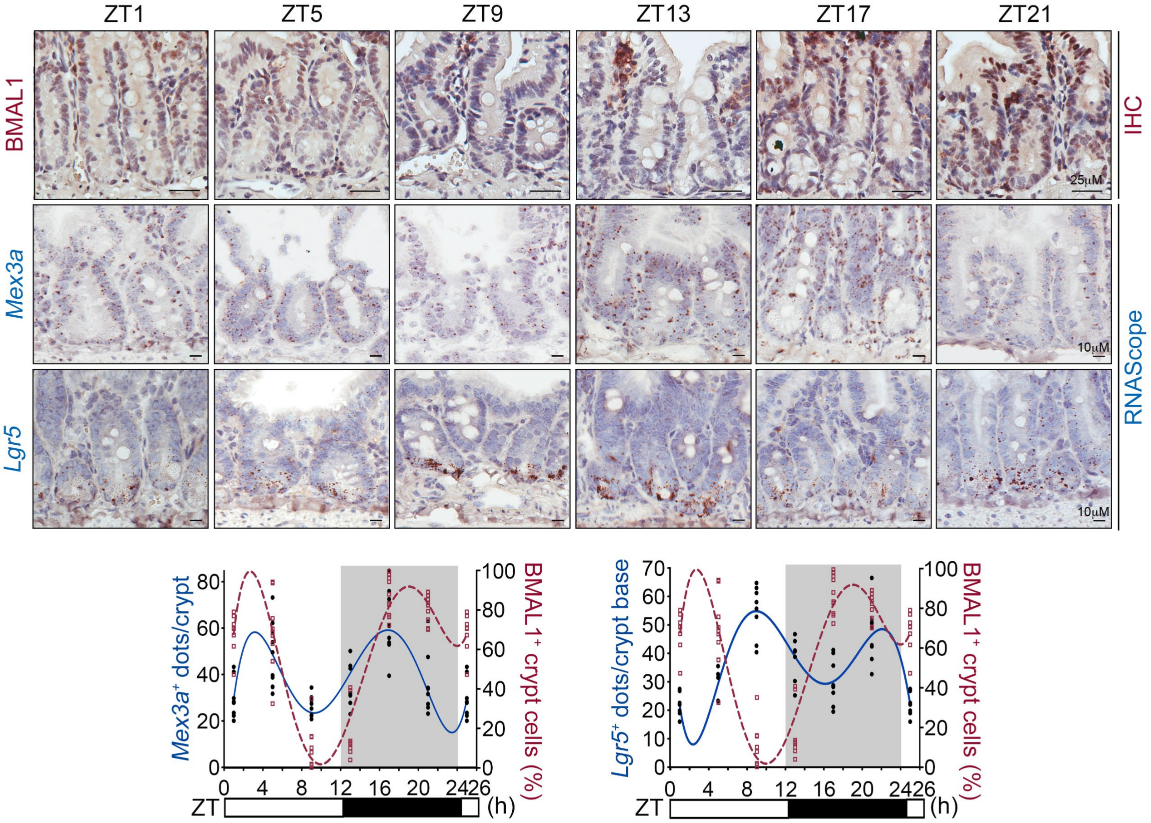

Fig S2. **Oscillation of BMAL1 protein, *Mex3a* and *Lgr5* mRNA in duodenal crypts of C57BL/6J mice.**

Top: Representative images of IHC staining to detect BMAL1 protein and RNAscope of *Mex3a* and *Lgr5* mRNA levels in WT B6 duodenum at the indicated ZT time points. Scale bars, 10 µM (n ≧ 2 mice per time point). Bottom: Quantification of IHC and RNAscope analysis of BMAL1, *Mex3a*, and *Lgr5* expression in B6 crypt bottom. The prevalence of $BMAL1^+$ cells in crypt bottom from IHC analysis (red dashed line) was quantified using the QuPath software. Cells with a mean DAB OD value greater than 0.2 are classified as $BMAL1^+$ cells. Quantification of *Mex3a* and *Lgr5* mRNA positive dots (blue line) was performed using the trainable weka segmentation classifier in Fiji software (n = ≧ 2 mice per time point, ≥ 50 crypts/n).

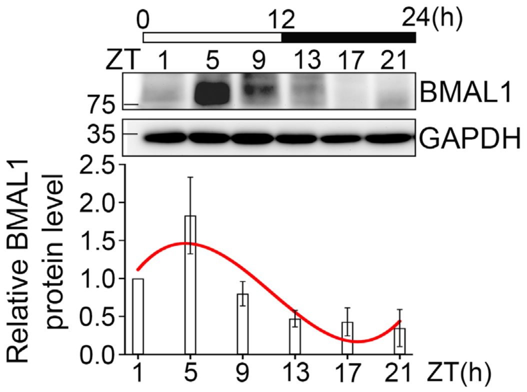

Fig S3. Oscillation of BMAL1 protein in duodenum of C57BL/6J mice.
Immunoblot of BMAL1 expression in B6 duodenum harvested every 4 hours under 12h:12h light dark cycle. GAPDH was used as a loading control. Blots shown are from one representative experiment of two independent experiments. The original blots and additional experiments with similar results are shown in this "Supplementary information" file (see below).

Table S1. Antibodies.

Antibody	Source	Cat#
Mouse monoclonal anti-Bmal1 (clone B-1)	Santa Cruz	sc-365645
Rabbit polyclonal anti-Mex3a	Abcam	ab79046
Chicken polyclonal anti-GFP	Abcam	ab13970
Rabbit polyclonal anti-GPX4	Cell Signaling	524554
Rabbit monoclonal anti-SOD1	Cell Signaling	37385
Rabbit monoclonal anti-xCT (SL7A11)	Abcam	ab175186
Rabbit polyclonal anti-Clock	Bethyl Laboratories, Inc.	A302-618A
Mouse monoclonal anti-p300 CT (clone RW128)	Millipore	05-257
Rabbit polyclonal anti-JARID1A/RBP2	Bethyl Laboratories, Inc.	A300-897A
Mouse monoclonal anti-BrdU (clone 3D4)	BD Bioscience	555627
Mouse monoclonal anti-GAPDH (clone GT239)	GeneTex	GTX627408
Rabbit polyclonal anti-Bmal1	Bethyl Laboratories, Inc.	A302-616A
Mouse monoclonal anti-Bmi1 (clone F6)	Abcam	ab14389
Rabbit polyclonal anti-Lgr5	GeneTex	GTX130204
Rabbit IgG isotype control	R&D Systems	AB-105-C
Mouse IgG1 isotype control	R&D Systems	MAB002R
Donkey anti-Chicken IgY (H+L), CF™ 488A	Sigma-Aldrich	SAB4600031
Donkey anti-Mouse IgG (H+L), CF™ 488A	Sigma-Aldrich	SAB4600035
Donkey anti-Mouse IgG (H+L), CF™ 647	Sigma-Aldrich	SAB4600176
Donkey anti-Rabbit IgG (H+L), CF™ 647	Sigma-Aldrich	SAB4600177
WestVision™ Peroxidase Polymer, Anti-Rabbit IgG	Vector Laboratories	WB-1000
WestVision™ Peroxidase Polymer, Anti-Mouse IgG	Vector Laboratories	WB-2000
Goat Anti-rabbit HRP antibody	Jackson ImmunoResearch	111-035-003
Goat Anti-mouse HRP antibody	Jackson ImmunoResearch	115-035-003

Table S2. Primer sequence for ChIP analysis.

Promoter/Site	Forward Primer	Reverse Primer
***Mex3a*/Region1 (-830~-403)**	GTCTGCTCCACCAGCTCTCT	CTTCCAGCGCTCAAACTCTT
***Mex3a*/Region2 (-1111~-831)**	TGGCTTCCCGGATCAGCCCC	AAAGCCCGCGGCCGCTACAG
***Mex3a*/FSC (far site control)**	CCCAGGAGTCCCCCACTTAC	TGGCACTTCTCTAGCCCTGC

Table S3. Probe sequence for RNA pull-down assays.

Gene/probe no.	Forward Primer	Reverse Primer
mGapdh/	TAATACGACTCACTATAGGGGGAGAG TGTTTCCTCGTCCCGTAGA	TATTTCTCGTGGTTCACACCCATCA
***Lgr5*/P1**	TAATACGACTCACTATAGGGAGAGAGC GTGCGAGCGGAGATGCTGC	CAGTGTGATGGGCAGCCCCGCG
***Lgr5*/P2**	TAATACGACTCACTATAGGGAGAGTCA CTGTGAGCTGGATGGCAGGA	GTCATCTAGCCACAGGTGCCTC
***Lgr5*/P3**	TAATACGACTCACTATAGGGAGAAATG CTCTCACAGACGTCCCTGT	GCAAATGCTGAAAAGCAGATAC
***Lgr5*/P4**	TAATACGACTCACTATAGGGAGACTGA ACTAAGAACACTGACTTTG	GGGAAGGACGACAGGAGATTGG
***Lgr5*/P5**	TAATACGACTAACTATAGGGTGTGACT GGGTTACATGGTTTAA	CGCCAGTACTGCCGTGGTCCAC
***Lgr5*/P6**	TAATACGACTCACTATAGGGAGAGCTT TCCTGCAATGCCTTGGTGGC	TACTGCCTCCTAGCAAGGGGAT
***Lgr5*/P7**	TAATACGACTCACTATAGGGAGAAGTA CAATGCCTCTCCCCTCTGC	GTATGCTTTCCCAGGCTGCCCA
***Lgr5*/P8**	TAATACGACTCACTATAGGGAGACCGT TTCTGGATGAGATCAAAAC	CTGAACAAATGATACAAGCGGT
***Lgr5*/P9**	TAATACGACTCACTATAGGGAGAGCTA AGAAGGAAAGCCATCAAGTC	CAAGTGCTGGGATTAAAGGCGC
***Lgr5*/P10**	TAATACGACTCACTATAGGGAGAGGGA GGCAGAGGCAGGTGGATTTC	GAAATAAATACAGATCCGACAA
***Lgr5*/P11**	TAATACGACTCACTATAGGGAGATGAG TAGGAGCATGCTTCTGGCA	TATATAAAAATAAATCTACCTA

Figure 2E (results in the manuscript and repeats)

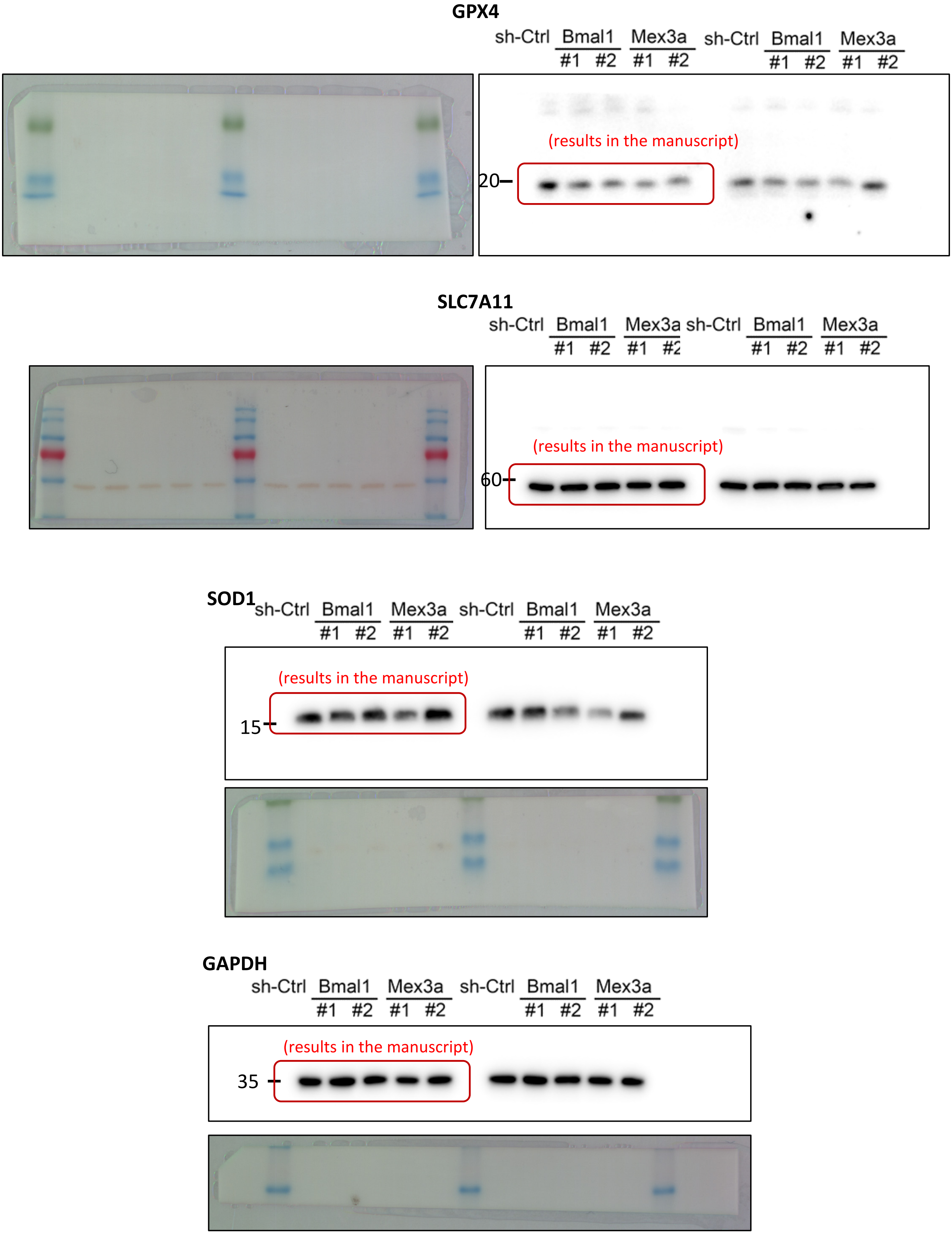

Figure 3A (results in the manuscript)

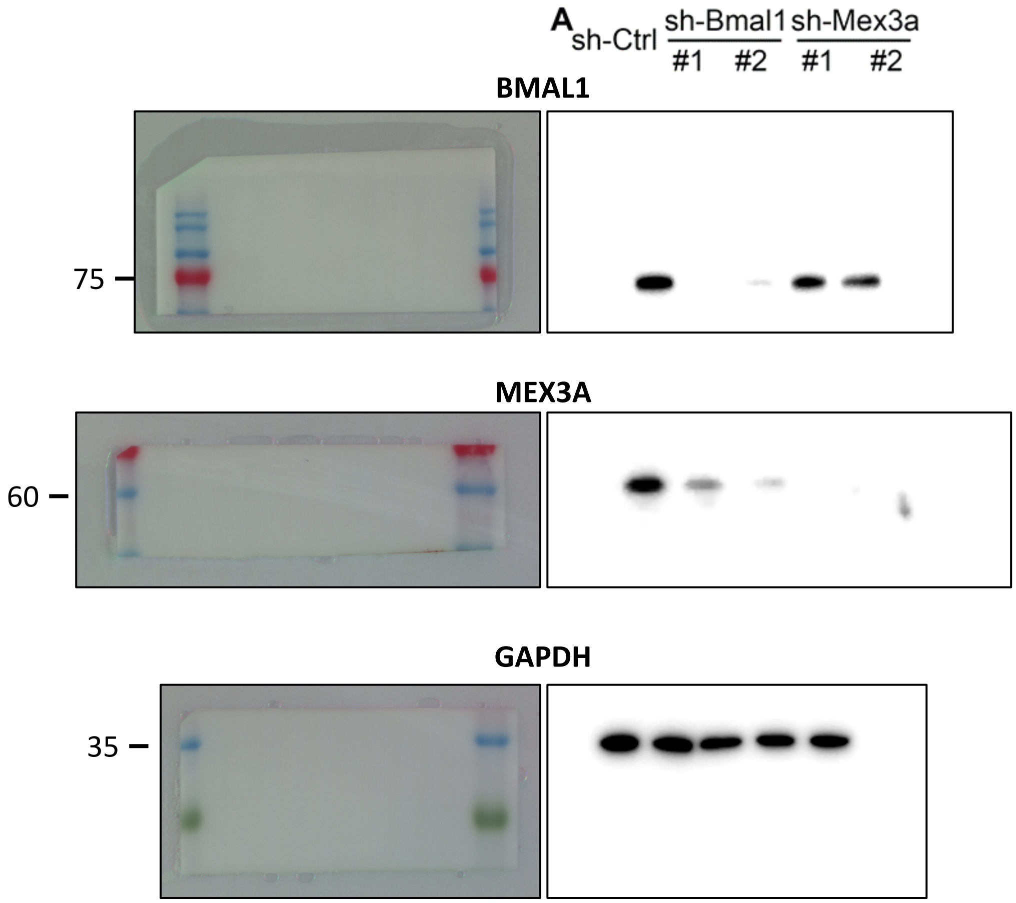

Figure 3A repeats, Figure 3D repeats

BMAL1

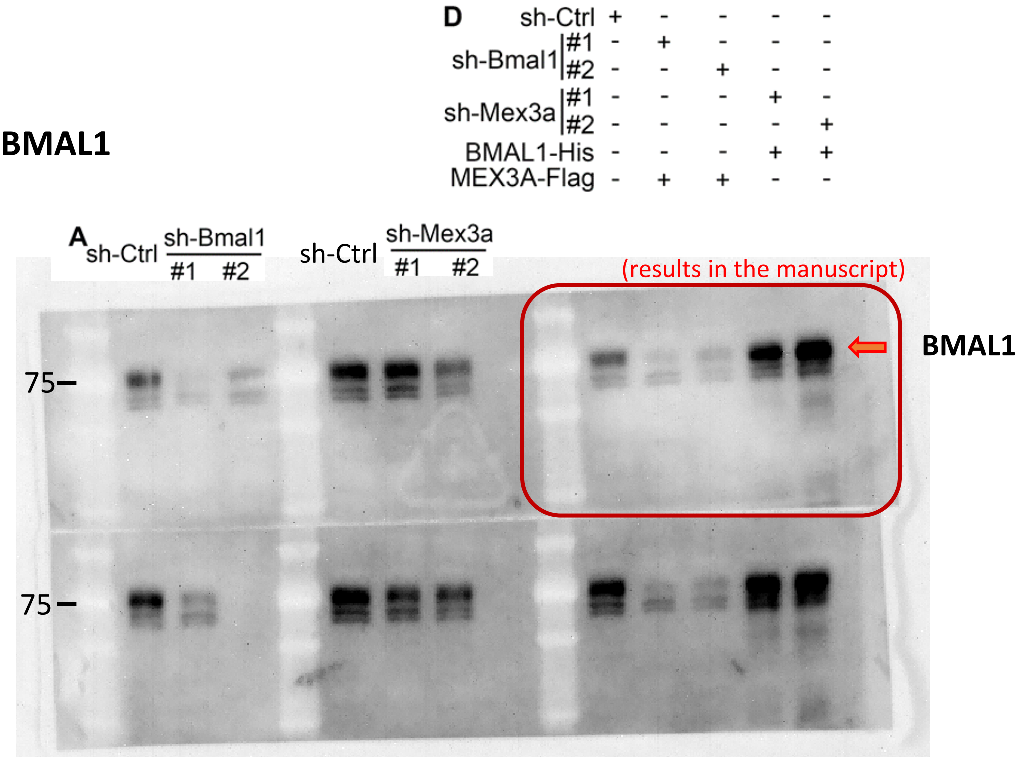

MEX3A

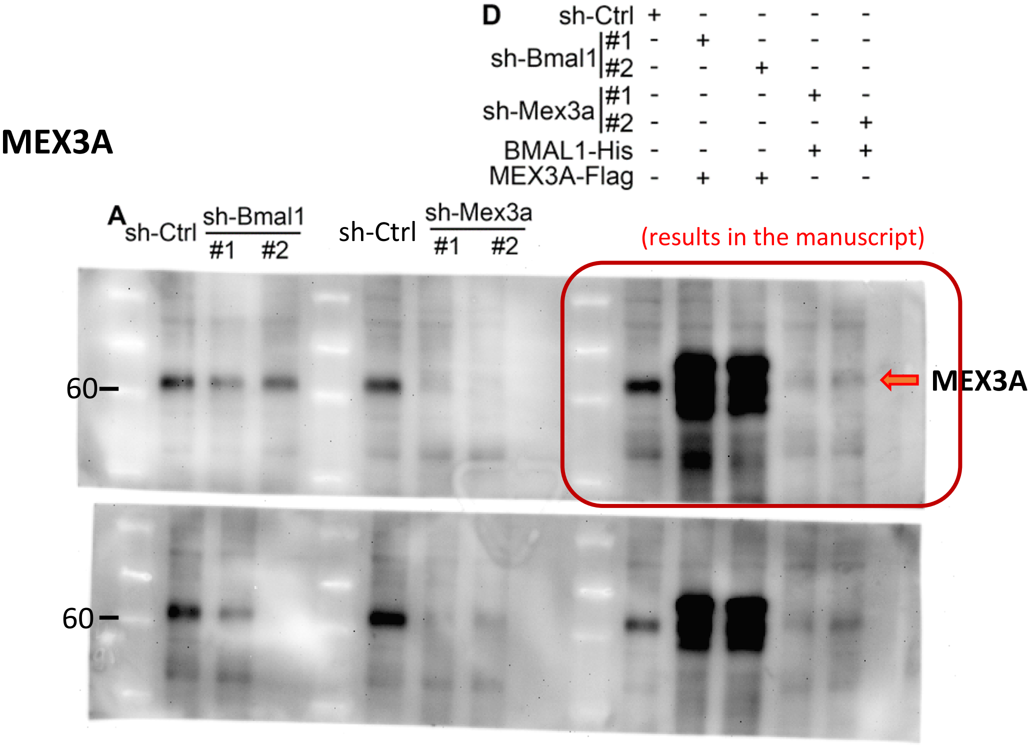

Figure 3B, 3C, 3D repeats

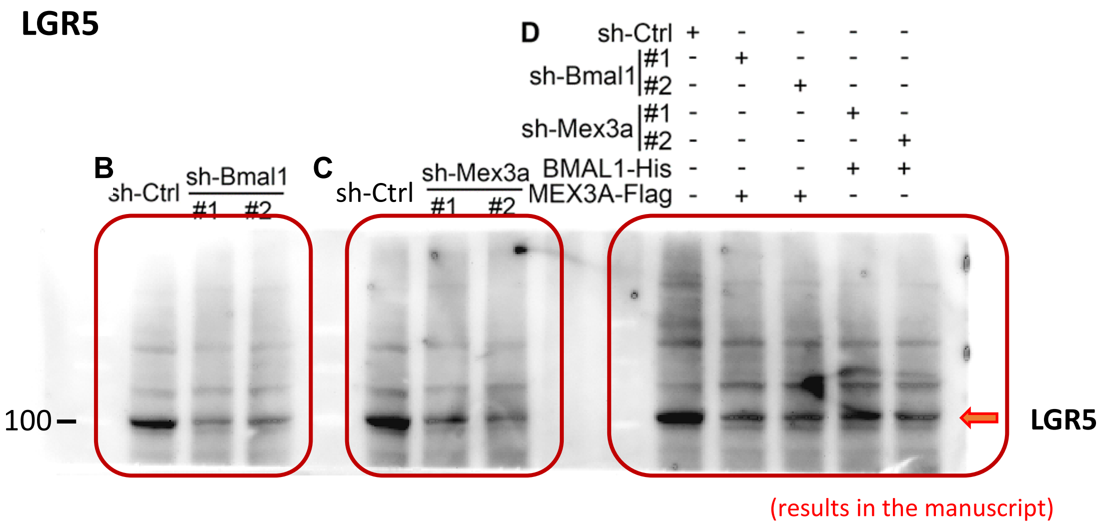

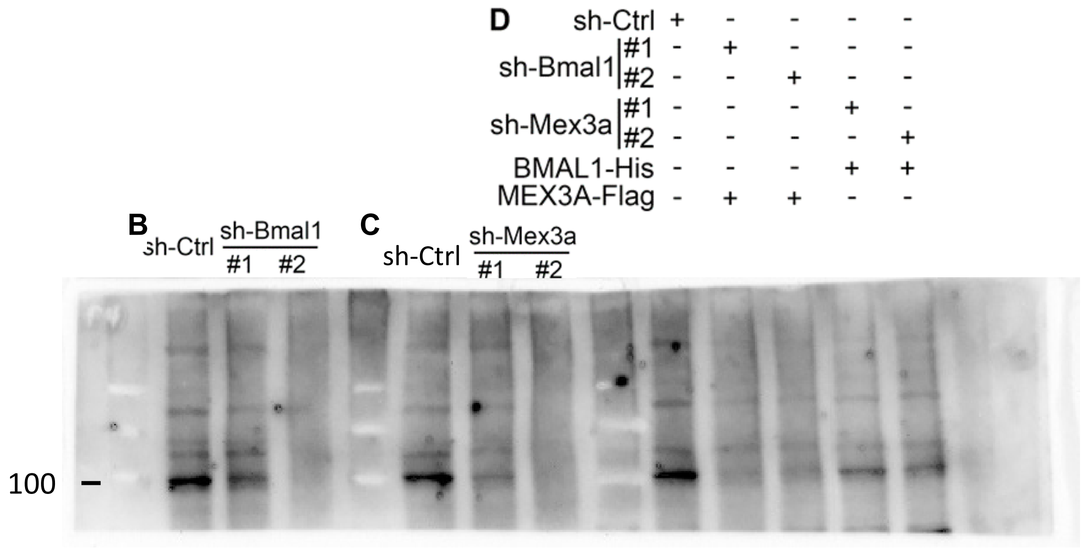

Figure 3B, 3C, 3D repeats

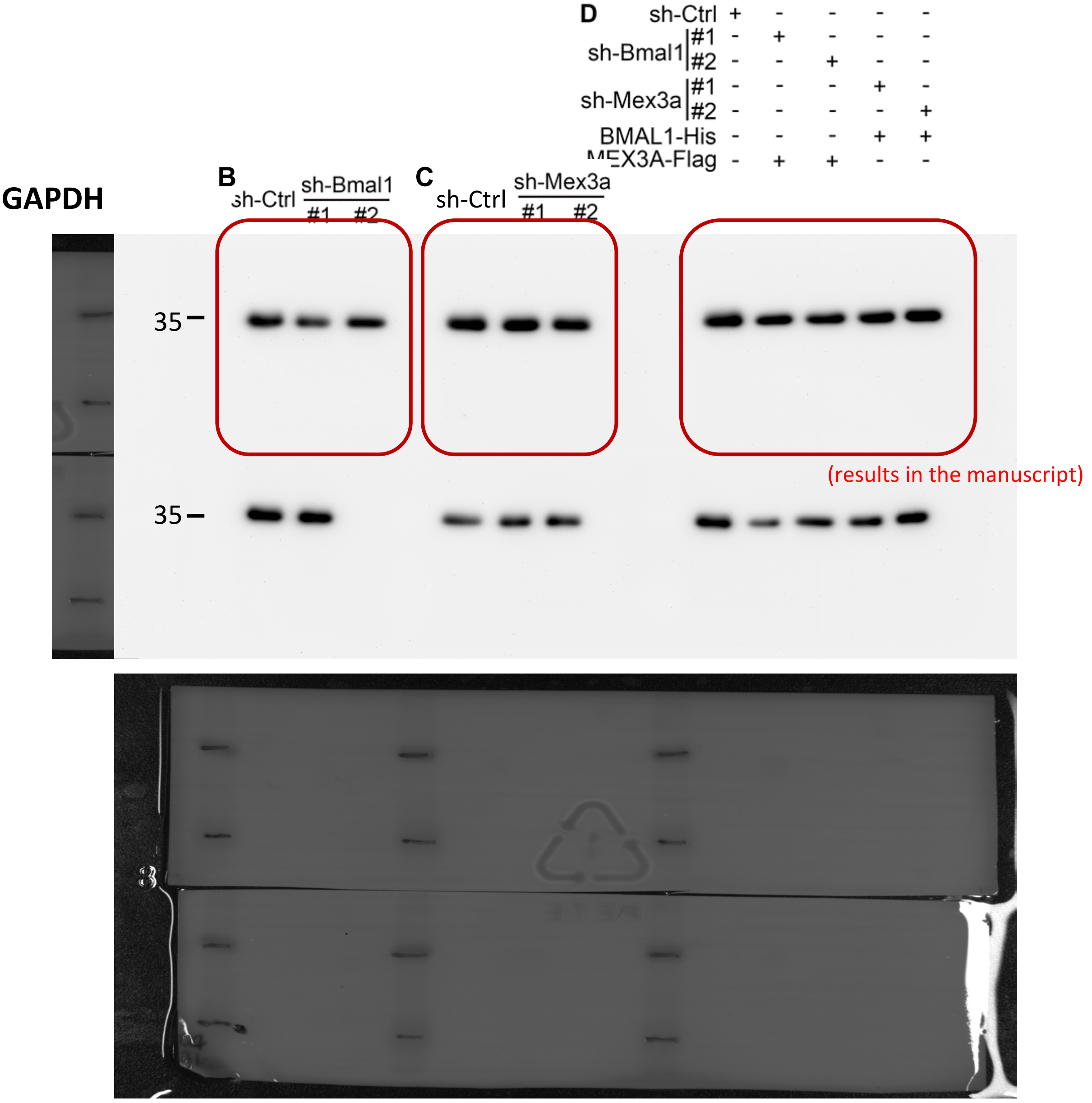

Figure 4C repeats

ChIP: BMAL1 on Mex3a P1 (-1111 ~ -831)

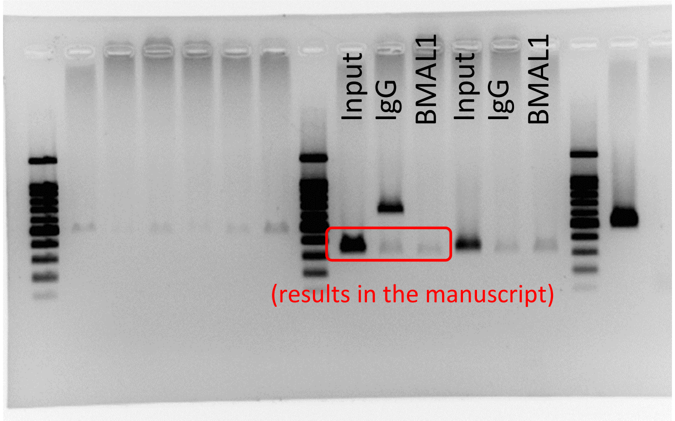

ChIP: BMAL1 on Mex3a P2 (-830 ~ -403)

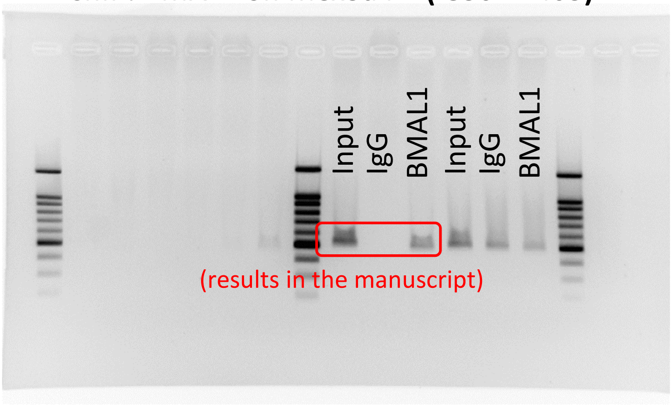

ChIP: BMAL1 on Mex3aP control (Chr 3, 88,521,891-88,522,294)

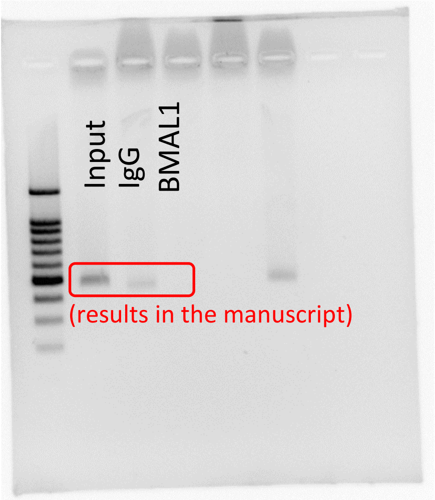

Figure 4D

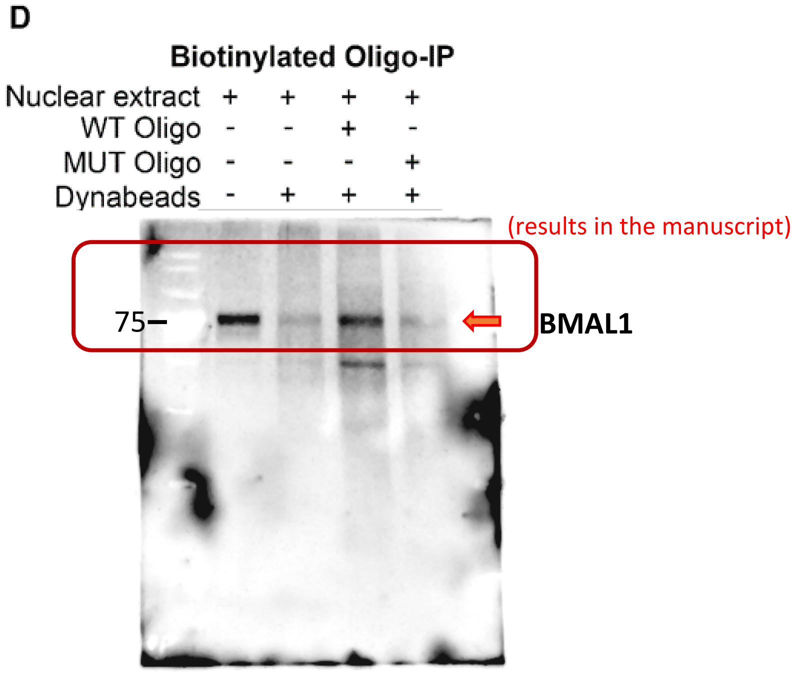
D
Biotinylated Oligo-IP
Nuclear extract + + + +
WT Oligo - - + -
MUT Oligo - - - +
Dynabeads - + + +
(results in the manuscript)
75
BMAL1

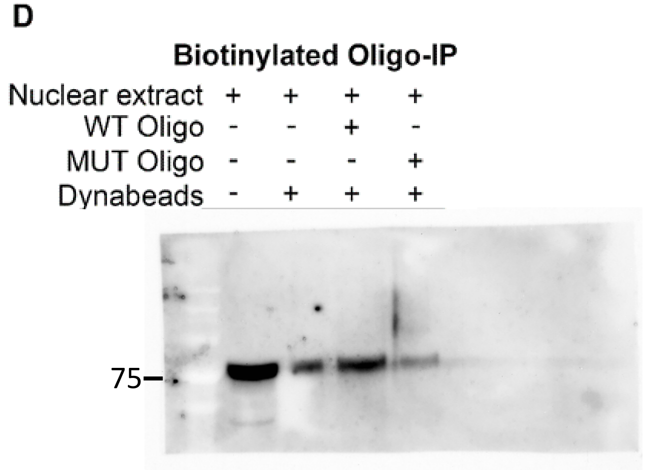
D
Biotinylated Oligo-IP
Nuclear extract + + + +
WT Oligo - - + -
MUT Oligo - - - +
Dynabeads - + + +
75

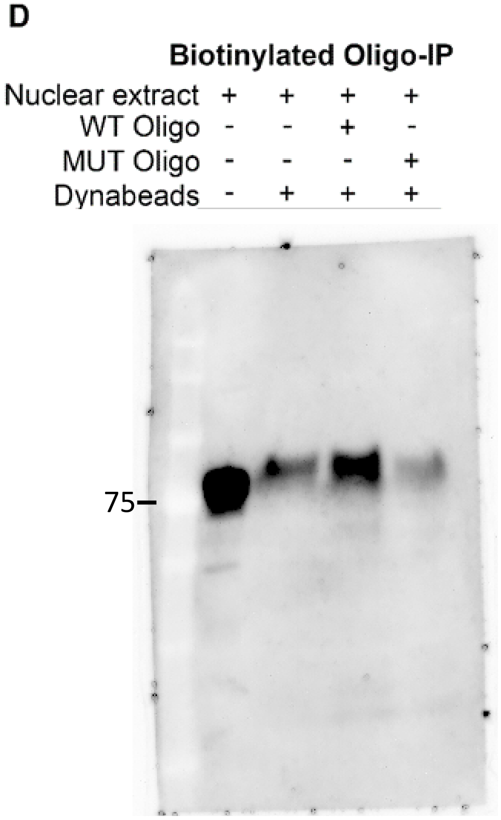
D
Biotinylated Oligo-IP
Nuclear extract + + + +
WT Oligo - - + -
MUT Oligo - - - +
Dynabeads - + + +
75

Figure 4F repeats

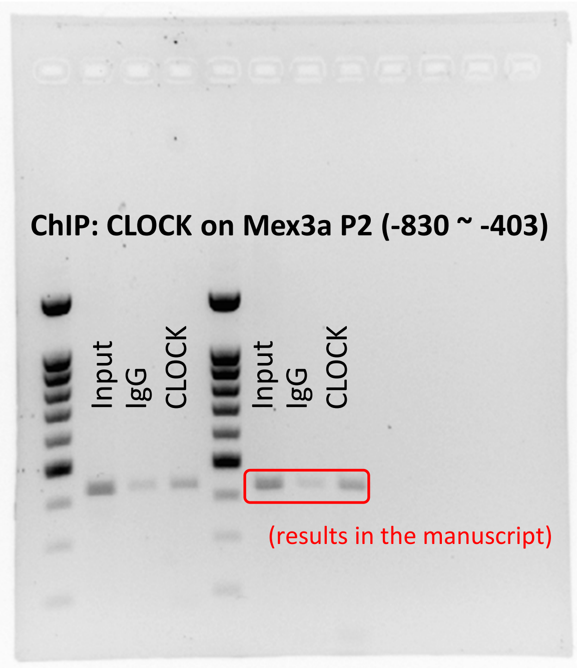

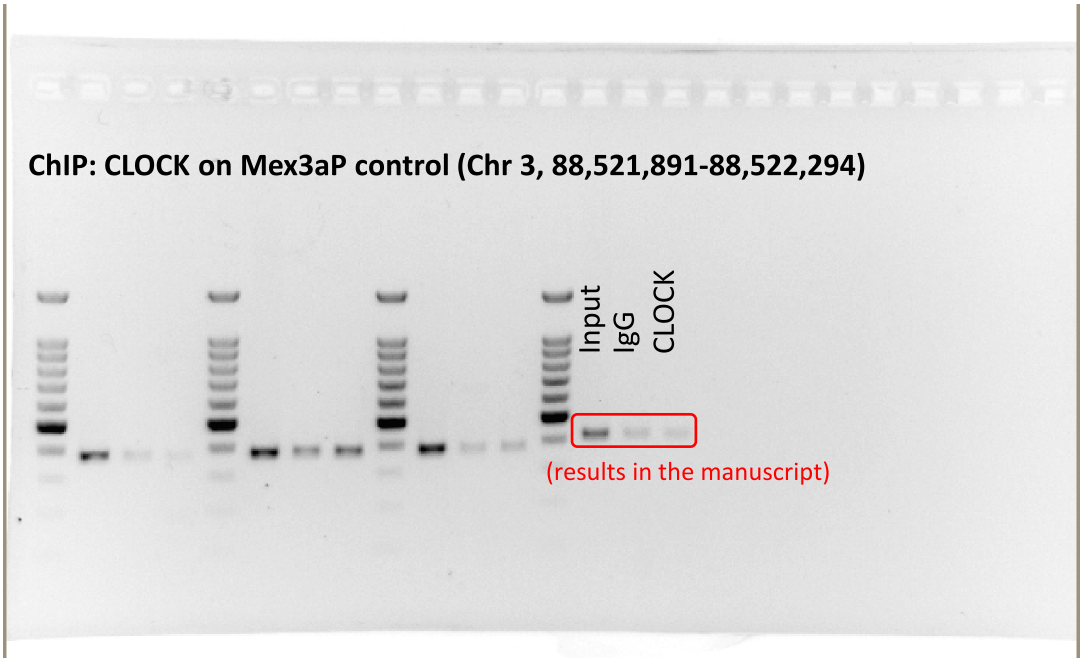

Figure 4G repeats

ChIP: JARID1A on Mex3a P2 (-830 ~ -403)

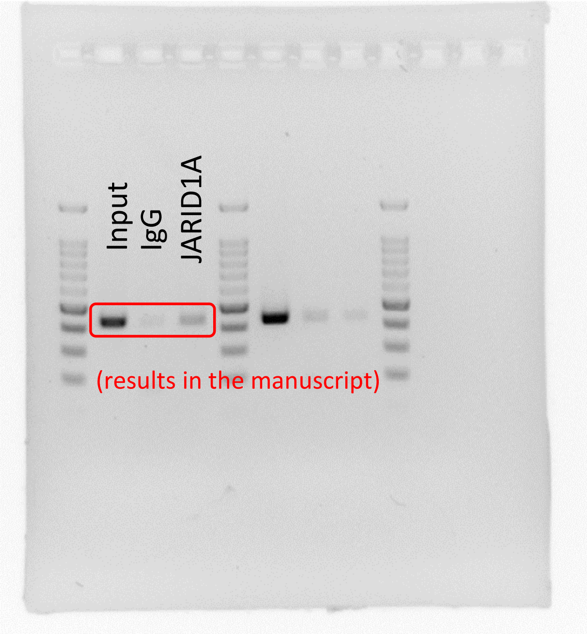

ChIP: JARID1A on Mex3a P2 (-830 ~ -403)

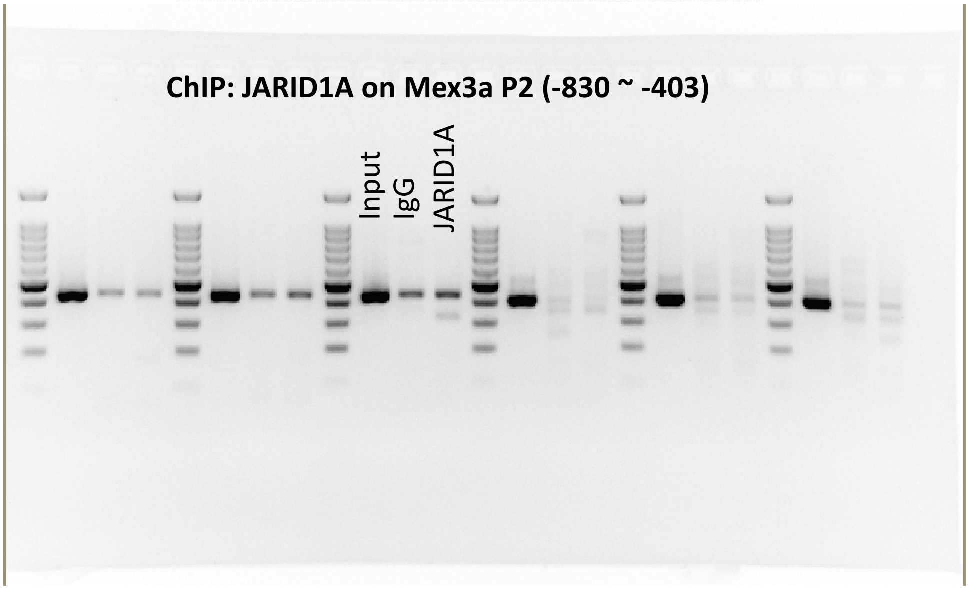

ChIP: JARID1A on Mex3aP control (Chr 3, 88,521,891-88,522,294)

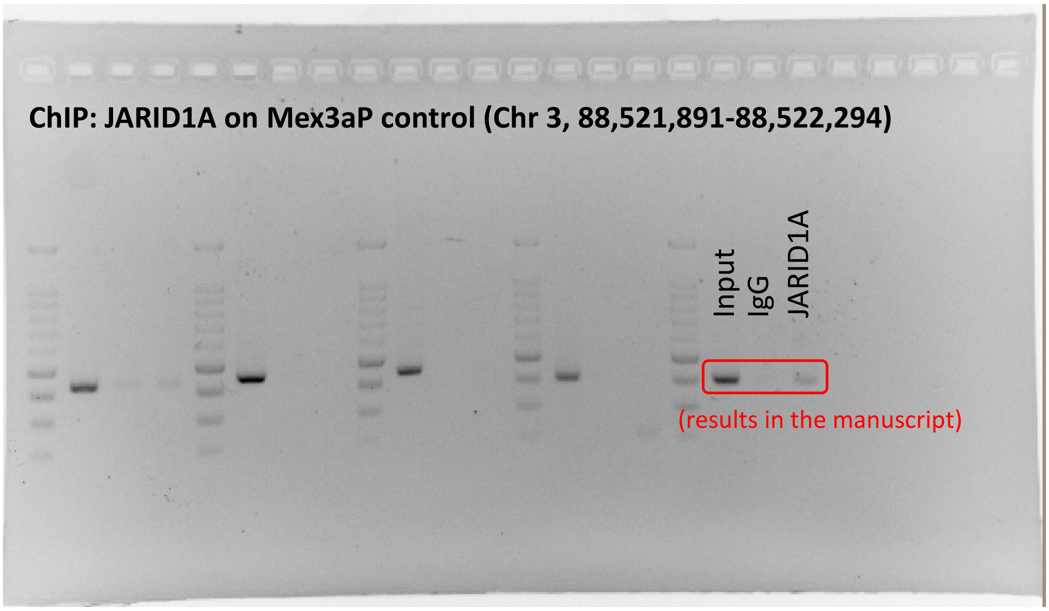

Figure 4H repeats

ChIP: p300 on Mex3a P2 (-830 ~ -403)

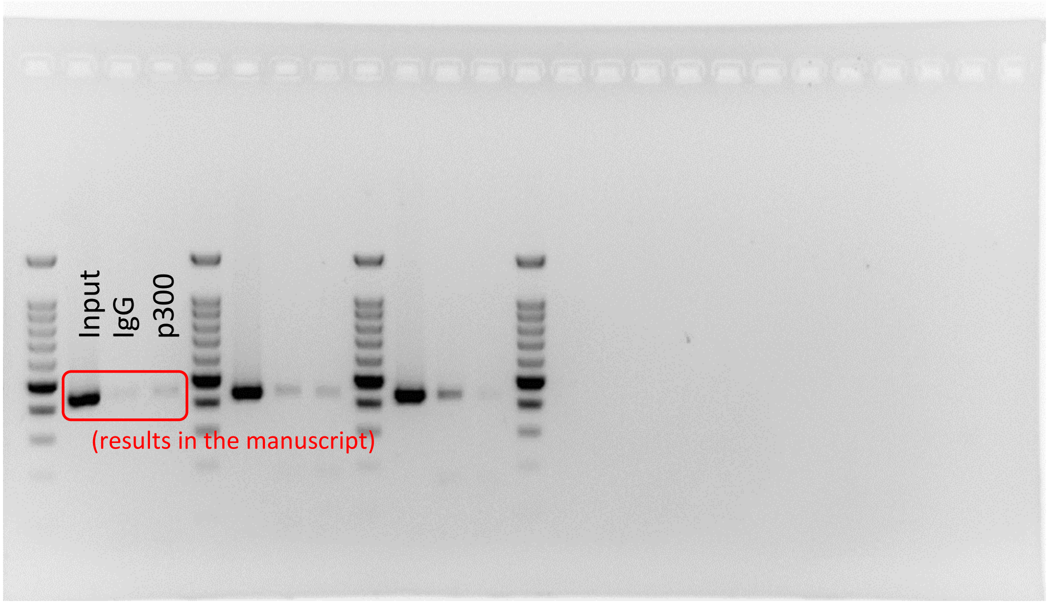

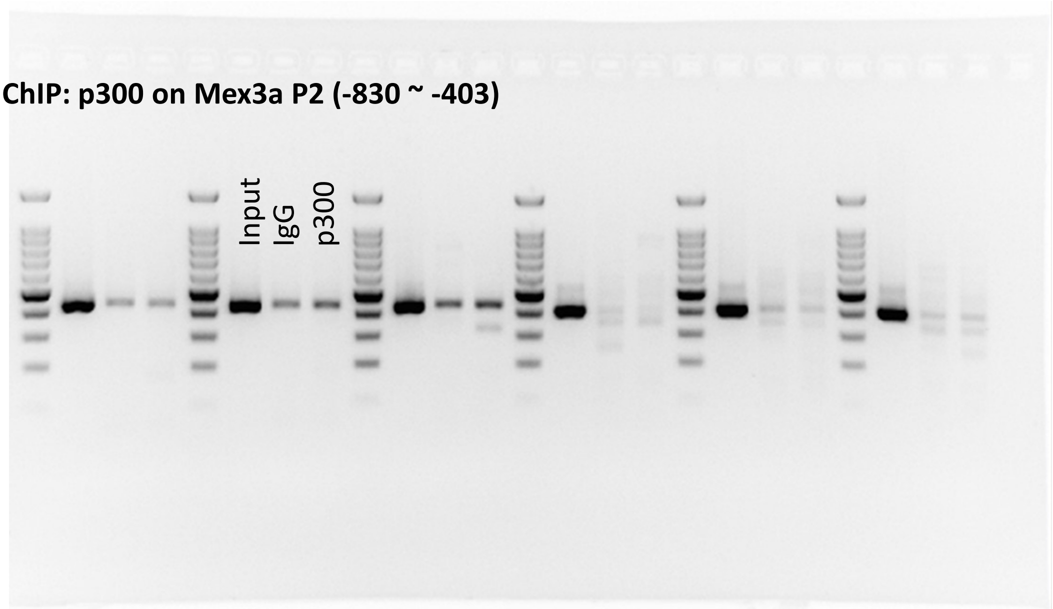

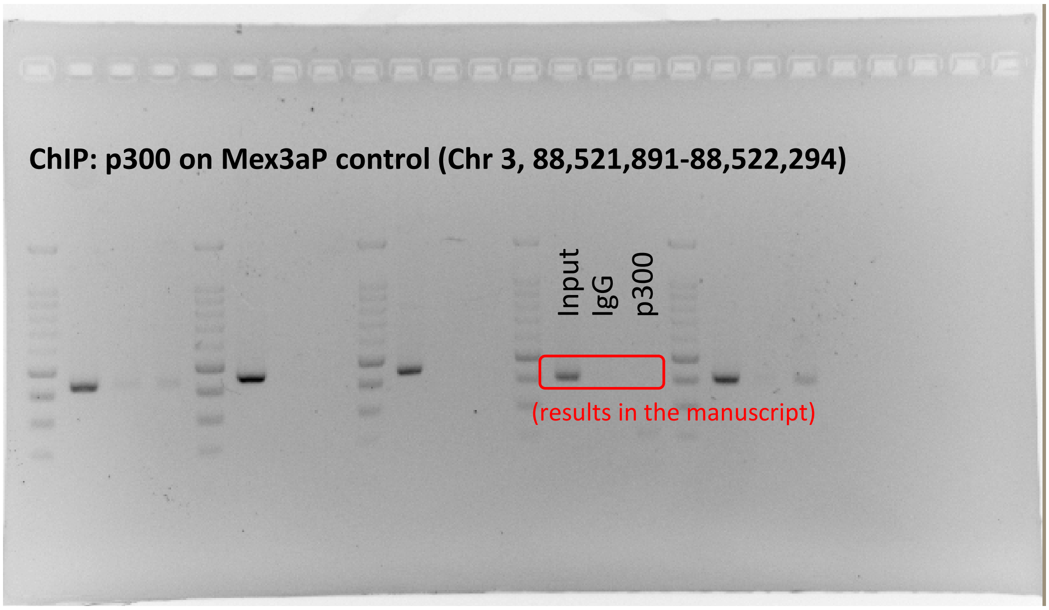

Figure 4J

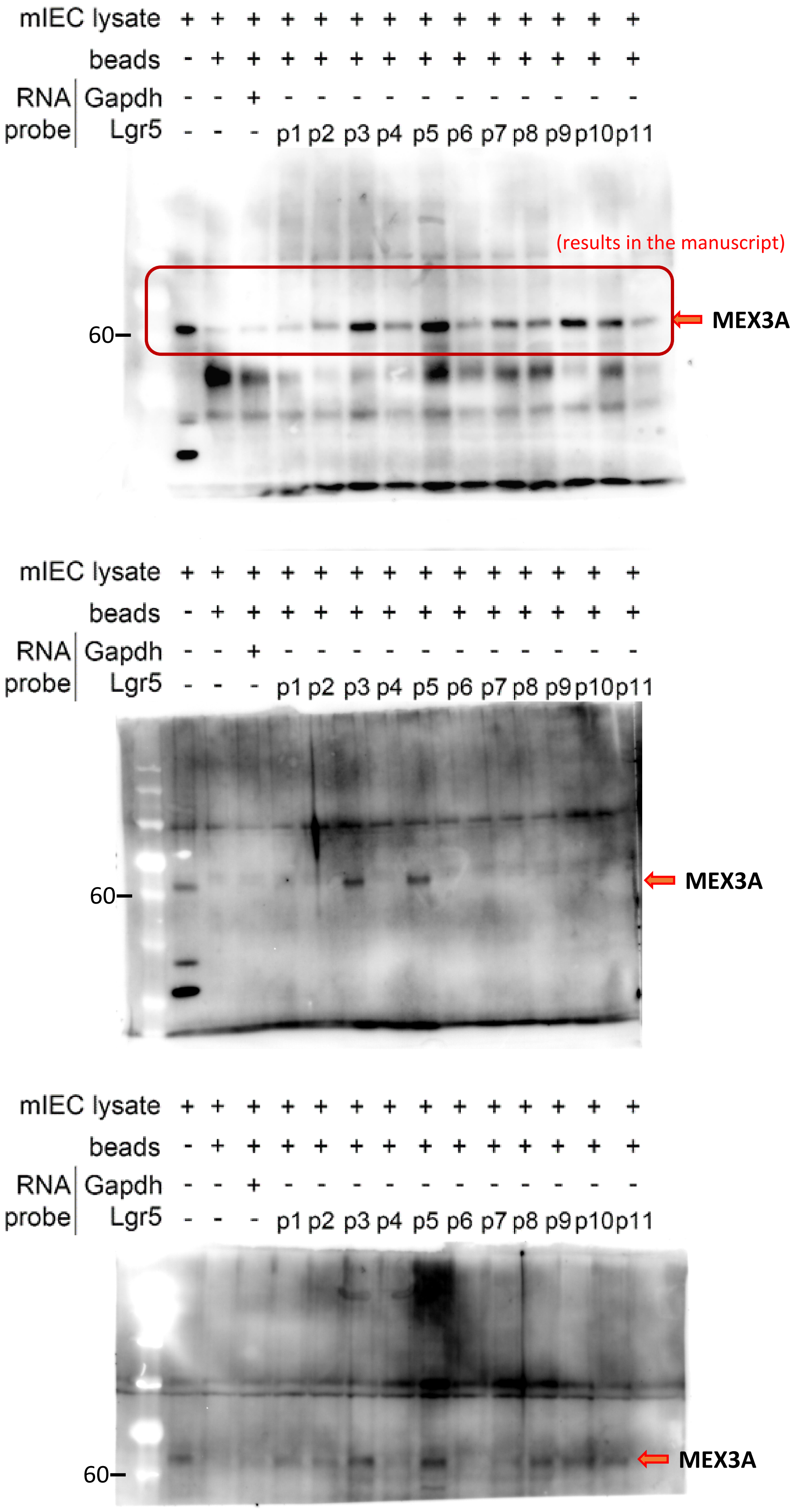
mIEC lysate + + + + + + + + + + + + + +
beads - + + + + + + + + + + + + +
RNA probe
Gapdh - - + - - - - - - - - - - -
Lgr5 - - - p1 p2 p3 p4 p5 p6 p7 p8 p9 p10 p11
(results in the manuscript)
60
MEX3A
mIEC lysate + + + + + + + + + + + + + +
beads - + + + + + + + + + + + + +
RNA probe
Gapdh - - + - - - - - - - - - - -
Lgr5 - - - p1 p2 p3 p4 p5 p6 p7 p8 p9 p10 p11
60
MEX3A
mIEC lysate + + + + + + + + + + + + + +
beads - + + + + + + + + + + + + +
RNA probe
Gapdh - - + - - - - - - - - - - -
Lgr5 - - - p1 p2 p3 p4 p5 p6 p7 p8 p9 p10 p11
60
MEX3A

Figure S1 (results in the manuscript and repeats)

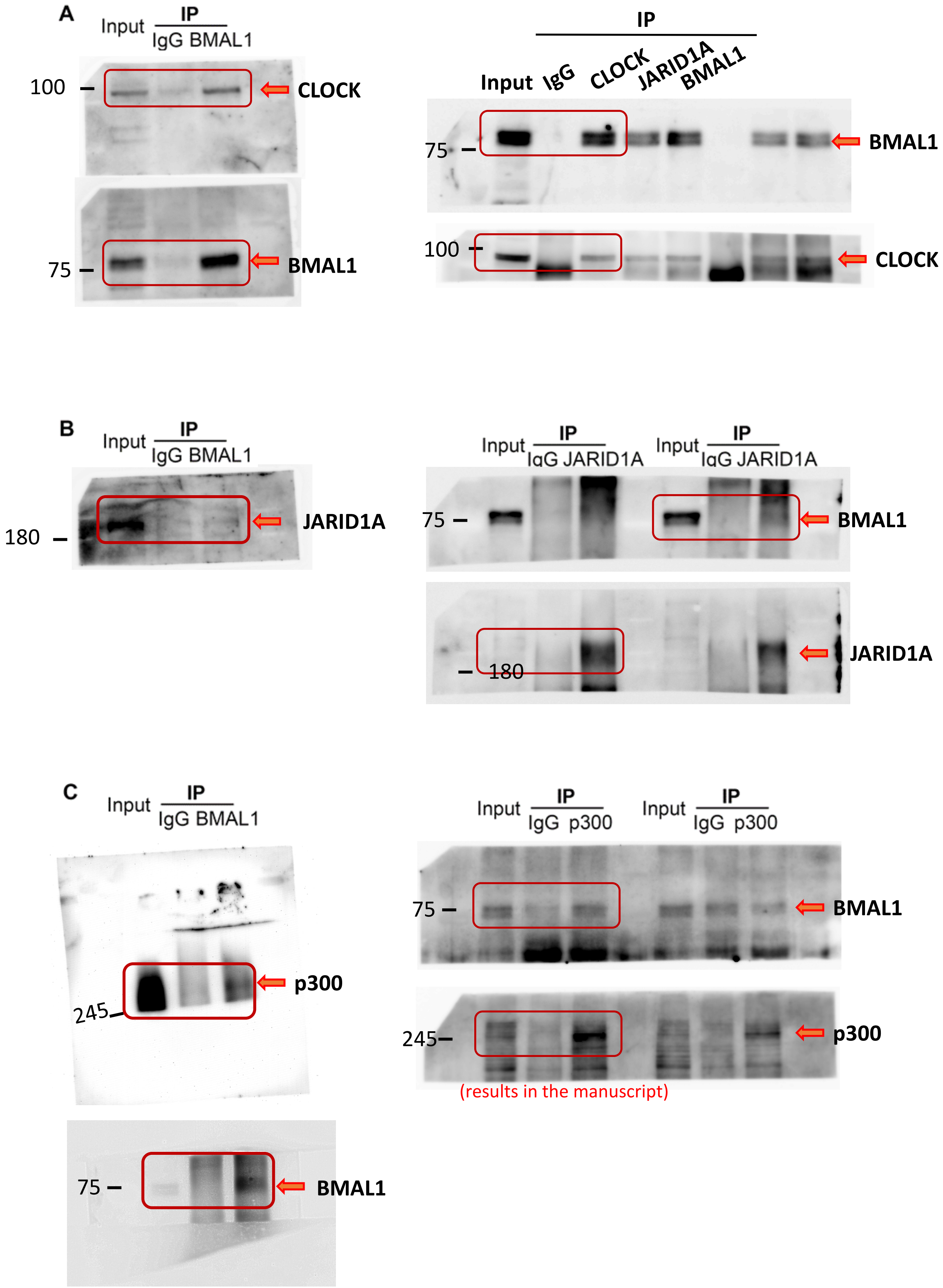

Figure S3 (results in the manuscript and repeats)

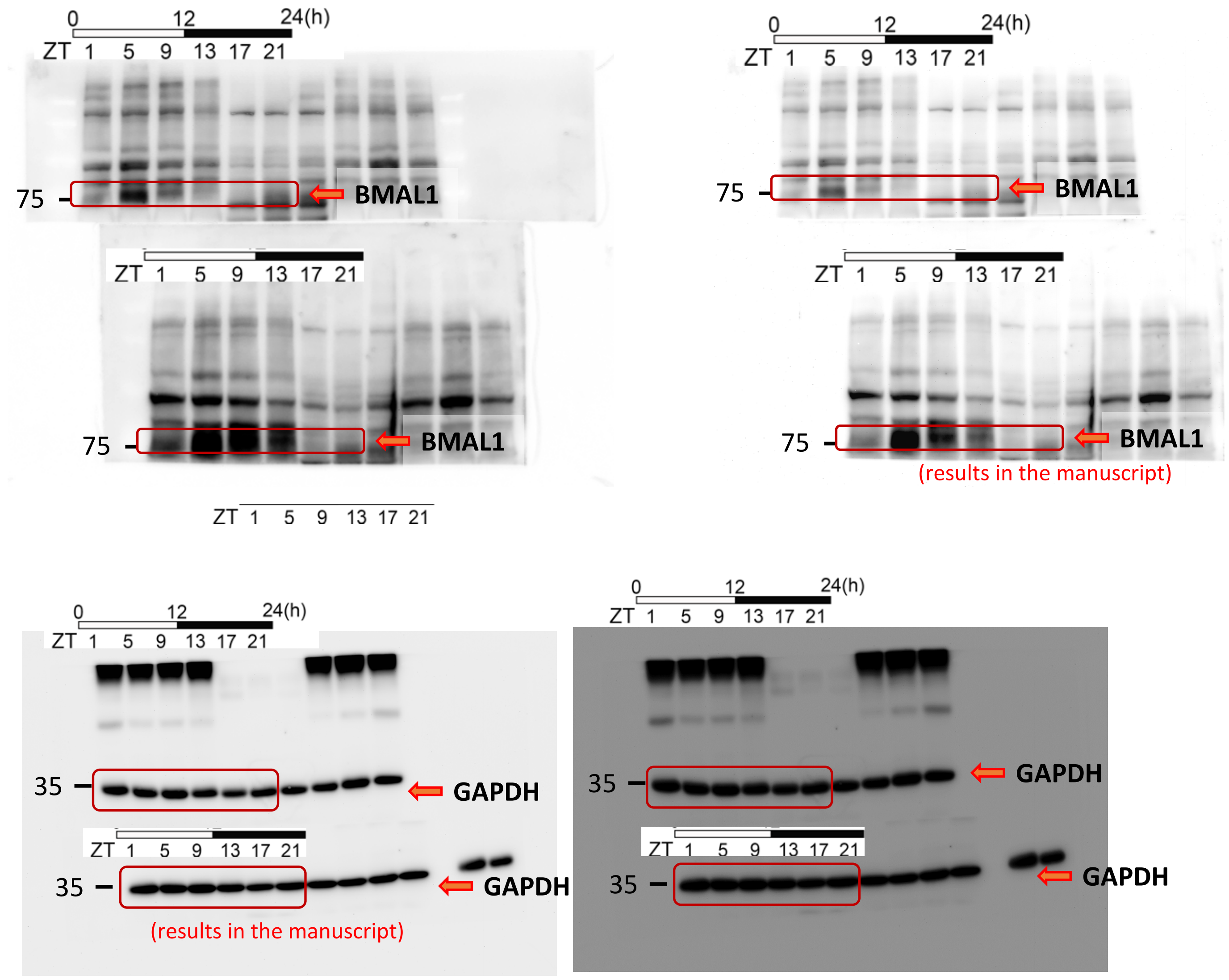

Supplement: Supplementary file 1 — Supplementary Information. [file 41598_2023_44997_MOESM1_ESM.pdf]
